# *ENB1* encodes a cellulose synthase 5 that directs synthesis of cell wall ingrowths in maize basal endosperm transfer cells

**DOI:** 10.1093/plcell/koab312

**Published:** 2021-12-22

**Authors:** Qun Wang, Mingmin Wang, Jian Chen, Weiwei Qi, Jinsheng Lai, Zeyang Ma, Rentao Song

**Affiliations:** State Key Laboratory of Plant Physiology and Biochemistry, National Maize Improvement Center, Beijing Key Laboratory of Crop Genetic Improvement, Joint International Research Laboratory of Crop Molecular Breeding, College of Agronomy and Biotechnology, China Agricultural University, Beijing 100193, China; Shanghai Key Laboratory of Bio-Energy Crops, Plant Science Center, School of Life Sciences, Shanghai University, Shanghai 200444, China; State Key Laboratory of Plant Physiology and Biochemistry, National Maize Improvement Center, Beijing Key Laboratory of Crop Genetic Improvement, Joint International Research Laboratory of Crop Molecular Breeding, College of Agronomy and Biotechnology, China Agricultural University, Beijing 100193, China; Shanghai Key Laboratory of Bio-Energy Crops, Plant Science Center, School of Life Sciences, Shanghai University, Shanghai 200444, China; State Key Laboratory of Plant Physiology and Biochemistry, National Maize Improvement Center, Beijing Key Laboratory of Crop Genetic Improvement, Joint International Research Laboratory of Crop Molecular Breeding, College of Agronomy and Biotechnology, China Agricultural University, Beijing 100193, China; State Key Laboratory of Plant Physiology and Biochemistry, National Maize Improvement Center, Beijing Key Laboratory of Crop Genetic Improvement, Joint International Research Laboratory of Crop Molecular Breeding, College of Agronomy and Biotechnology, China Agricultural University, Beijing 100193, China; State Key Laboratory of Plant Physiology and Biochemistry, National Maize Improvement Center, Beijing Key Laboratory of Crop Genetic Improvement, Joint International Research Laboratory of Crop Molecular Breeding, College of Agronomy and Biotechnology, China Agricultural University, Beijing 100193, China

## Abstract

Development of the endosperm is strikingly different in monocots and dicots: it often manifests as a persistent tissue in the former and transient tissue in the latter. Little is known about the controlling mechanisms responsible for these different outcomes. Here we characterized a maize (*Zea mays*) mutant, *endosperm breakdown1* (*enb1*), in which the typically persistent endosperm (PE) was drastically degraded during kernel development. *ENB1* encodes a cellulose synthase 5 that is predominantly expressed in the basal endosperm transfer layer (BETL) of endosperm cells. Loss of ENB1 function caused a drastic reduction in formation of flange cell wall ingrowths (ingrowths) in BETL cells. Defective ingrowths impair nutrient uptake, leading to premature utilization of endosperm starch to nourish the embryo. Similarly, developing wild-type kernels cultured in vitro with a low level of sucrose manifested early endosperm breakdown. *ENB1* expression is induced by sucrose via the BETL-specific Myb-Related Protein1 transcription factor. Overexpression of *ENB1* enhanced development of flange ingrowths, facilitating sucrose transport into BETL cells and increasing kernel weight. The results demonstrated that ENB1 enhances sucrose supply to the endosperm and contributes to a PE in the kernel.

## Teaser

Cell wall ingrowths of maize basal endosperm transfer layer cells are synthesized by a cellulose synthase complex containing ENB1 that enhances sucrose import, sustaining a persistent endosperm.

##  

**Figure:**
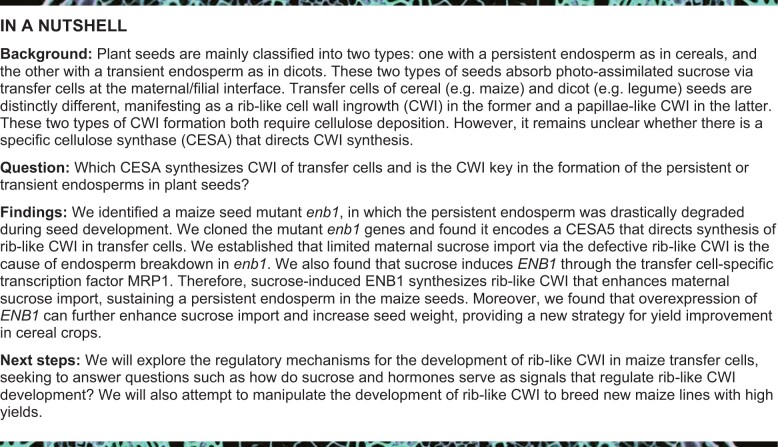


## Introduction

The angiosperm seed contains two zygotic components, the endosperm, and the embryo, and their development is distinctly different in monocots and dicots. Generally, in monocots such as maize (*Zea mays*), the endosperm is a persistent tissue that accumulates large amounts of nutrient reserves, primarily starch, and ultimately occupies the largest portion of the mature seed (Olsen, 2004; Sabelli and Larkins, 2009). In contrast, in dicots, such as Arabidopsis (*Arabidopsis thaliana*), the endosperm is a transient tissue and is largely consumed during embryo development, leaving one layer of cells at maturation (Li and Berger, 2012; Sreenivasulu and Wobus, 2013; Grimault et al., 2015). Although these developmental patterns are well-known, the underlying mechanisms are poorly understood.

There is broad agreement that maternal nutrients, including sucrose, are transported to seeds via transfer cells (Felker and Shannon, 1980; Patrick and Offler, 1995; Bagnall et al., 2000; Bihmidine et al., 2013; Chourey and Hueros, 2017). In maize, these are found in the basal endosperm transfer layer (BETL) of the endosperm, while in Arabidopsis they occur in the seed coat (Felker and Shannon, 1980; Bihmidine et al., 2013; Lafon-Placette and Kohler, 2014; Chen et al., 2015). Maize BETL cells contain a cell wall (CW)-bound invertase, MN1, that cleaves sucrose into hexoses (Cheng et al., 1996; Chourey and Hueros, 2017), and the SWEET4c transporter delivers them to inner starchy endosperm (SE) cells (Sosso et al., 2015) where they are stored as starch or transferred to the embryo for its development (Hannah and Boehlein, 2017; Rolletschek et al., 2017).

The nutrient transport capacity of transfer cells depends on unique CW ingrowths (CWI; ingrowths) that increase the surface area of the plasma membrane (PM), creating a high rate of transport flux (Pate and Gunning, 1972; Offler et al., 2003). Cytological observations have identified two different architectural types of ingrowths: flange-type and reticulate-type, which are characterized by ribs (or bars) and papillae-like wall ingrowths, respectively (Talbot et al., 2002; Offler et al., 2003). Formation of the ingrowths involves cellulose synthesis (Talbot et al., 2007a, 2007b; McCurdy et al., 2008) by the cellulose synthase (CESA) complex (CSC; McFarlane et al., 2014; Polko and Kieber, 2019). It is unclear if there is a specific CESA or CSC that mediates formation of wall ingrowths.

Here we characterized *endosperm breakdown1* (*enb1*), a novel maize mutant showing endosperm breakdown during kernel development. We report positional cloning of *enb1* and show it encodes a ZmCESA5. Cytological and biochemical analyses showed the nonfunctional ZmCESA5 decreased nutrient uptake from maternal tissue, creating a degraded endosperm (DE) in the kernel. Moreover, overexpression of wild-type (WT) ZmCESA5 increased kernel weight, suggesting it as a potential target for yield improvement.

## Results

### *enb1* triggers endosperm breakdown

**Figure 1:**
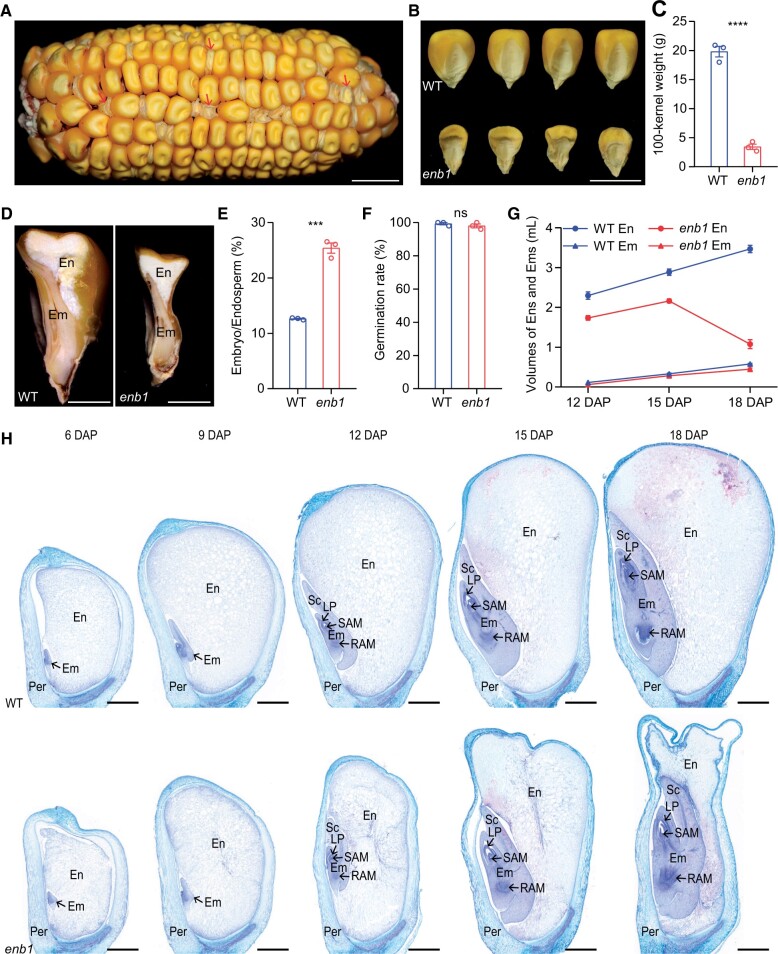
*enb1* triggers endosperm breakdown. A, Mature F2 ear of *enb1* × W22. Red arrows indicate the *enb1* kernels. Bar = 1 cm. B, Randomly selected mature kernels of WT and *enb1* in a segregated F2 population. Bar = 1 cm. C, 100-kernel weight of randomly selected mature WT and *enb1* kernels in a segregated F2 population. Data are mean ± standard error of the mean (sem, *n* = 3 biologically independent samples). ****P < 0.0001; Student’s *t* test. D, Longitudinal sections of mature WT and *enb1* kernels. En, endosperm; Em, embryo. Bar = 2 mm. E, Ratio of the dry weights of 10 embryos to the dry weights of the corresponding 10 endosperms of WT and *enb1* plants, respectively. Data are mean ± sem (*n* = 3 biologically independent samples). ***P < 0.001; Student’s *t* test. F, Germination rate of the WT and *enb1* kernels. Data are mean ± sem (*n* = 3 biologically independent samples). ns, not significant; Student’s *t* test. G, Volumes of 30 endosperms (Ens) and 30 embryos (Ems) in developing WT and *enb1* kernels. Data are mean ± sem (*n* = 3 biologically independent samples). H, Cytological observations of developing WT and *enb1* kernels from 6 to 18 DAP. These sections were stained with toluidine blue. En, endosperm; Em, embryo; Per, pericarp; LP, leaf primordia; SAM, shoot apical meristem; RAM, root apical meristem. Bar = 1 mm.

A defective kernel mutant stock named *5512K* was obtained from the Maize Genetics Cooperation Stock Center. This stock was crossed into the W22 inbred line, where F_2_ ears displayed 1:3 segregation for mutant versus WT kernels (Figure 1A; Supplemental Figure S1A), indicating that the defective kernel mutant is the result of a monogenic recessive mutation. The *5512K* kernels are small and shrunken (Figure 1, A and B), with only 17.3% of the WT 100-kernel weight (Figure 1C). Although the endosperm and the embryo of *5512K* were both affected (Figure 1D; Supplemental Figure S1B), the mutation more dramatically impacted the endosperm than the embryo (Figure 1E). Intriguingly, *5512K* kernels germinated normally and developed into normal fertile plants (Figure 1F; Supplemental Figure S1, C and D), indicating *5512K* mutant kernels are small but fully viable.

We carried out paraffin sectioning to observe the effect of the *5512K* mutation on kernel development between 6 and 18 days after pollination (DAP). In WT, both the endosperm and embryo enlarged progressively during kernel development (Figure 1, G and H). In mutant *5512K* kernels, the embryo also progressively enlarged, forming typical embryonic structures like the WT (Figure 1H). However, while the mutant endosperms enlarged between 6 and 15 DAP, they became strikingly depleted starting from 15 to 18 DAP (Figure 1, G and H). This indicated an endosperm breakdown process was occurring during *5512K* kernel development. Hence, we named *5512K* *enb1*.

### *ENB1* encodes a CESA5 in maize

**Figure 2:**
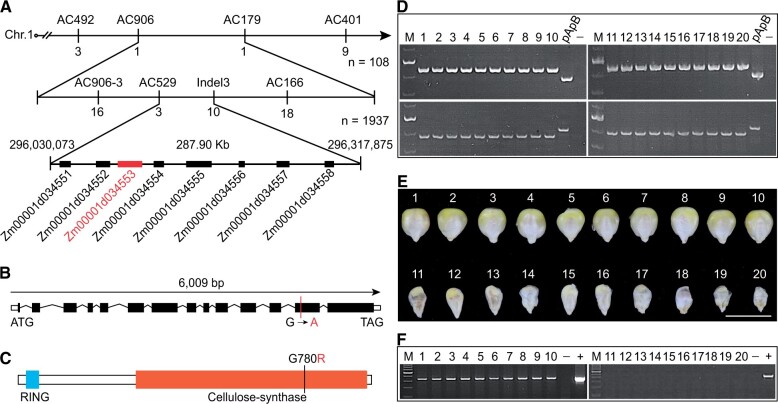
*Zm00001d034553* is *ENB1*. A, Positional cloning of *ENB1*. The *enb1* mutant was crossed into the W22 inbred line to produce the F1, and then the F1 selfed to generate the F2 mapping population. The *ENB1* locus was first mapped to a 9.11-Mb genomic interval on the long arm of chromosome 1 using the maizeSNP3072 genotyping array. Then the *ENB1* locus was placed between the molecular markers AC906 and AC179 using the mapping population of 108 individuals. Approximately 1,937 individuals were characterized, and the *ENB1* locus was eventually mapped to a 287.90-kb region between AC529 and Indel3. The number beneath each molecular marker indicates the recombinants between *enb1* and the molecular marker. Eight boxes represent eight predicted genes, and the candidate gene is in red. B, Gene model of *Zm00001d034553* and mutation site of *enb1*. Black boxes represent exons, black lines represent introns, and white boxes represent untranslated regions. C, Protein domains and mutation site of Zm00001d034553. D–F, Functional complementation test of *enb1*. D, The 9948-bp *Zm00001d034553* genomic DNA fragment containing the entire coding sequence, a 2685-bp upstream region of the start codon, and the 2,068-bp downstream region of the stop codon was transformed into the Hi-II hybrid pApB. Transgenic T0 lines were crossed into heterozygous plants (+/*enb1*), and then self-pollinated to obtain the F2 ears. Twenty representative kernels with homozygous *enb1* alleles were identified using the molecular markers AC906-3 and AC024, which are tightly linked to the *enb1* locus. pApB was used as the WT allele (*ENB1*) control; –, PCR blank control. E, Twenty representative kernels harboring homozygous *enb1* alleles exhibited WT (kernels 1–10) or mutant phenotypes (kernels 11–20). Bar = 1 cm. F, Transgenic detection of these above kernels using the Bar primer pairs. The kernels (1–10) harbored the transgene, while the kernels (11–20) did not harbor the transgene. –, pApB genomic DNA without transgene as a negative control; +, *Zm00001d034553* transgene construct as a positive control.

To investigate the gene responsible for the mutant phenotype of *enb1* kernels, we conducted positional cloning of *ENB1*. We mapped *ENB1* to a 287.90-kb genomic interval on chromosome 1 that contained eight predicted genes (Figure 2A; Supplemental Table S1). DNA sequencing of the predicted genes revealed *Zm00001d034553* contains a G-to-A mutation in exon 13 of the *enb1* allele (Figure 2B), causing an amino acid (aa) substitution (G780R) difference between WT and *enb1* alleles (Figure 2C). A transgenic functional complementation test was conducted using the genomic DNA fragment of *Zm00001d034553* driven by its native promoter. The *enb1* kernels harboring the transgene manifested the WT phenotype, while those without the transgene showed the mutant phenotype (Figure 2, D–F). Additionally, we used the clustered regularly interspersed short palindromic repeat (CRISPR)/CRISPR-associated protein9 (Cas9) editing system to generate loss-of-function mutants of *Zm00001d034553*, and found that the kernel phenotype of these mutants was similar to that of *enb1* (Supplemental Figure S2). This result confirmed that *Zm00001d034553* is *ENB1*.

**Figure 3:**
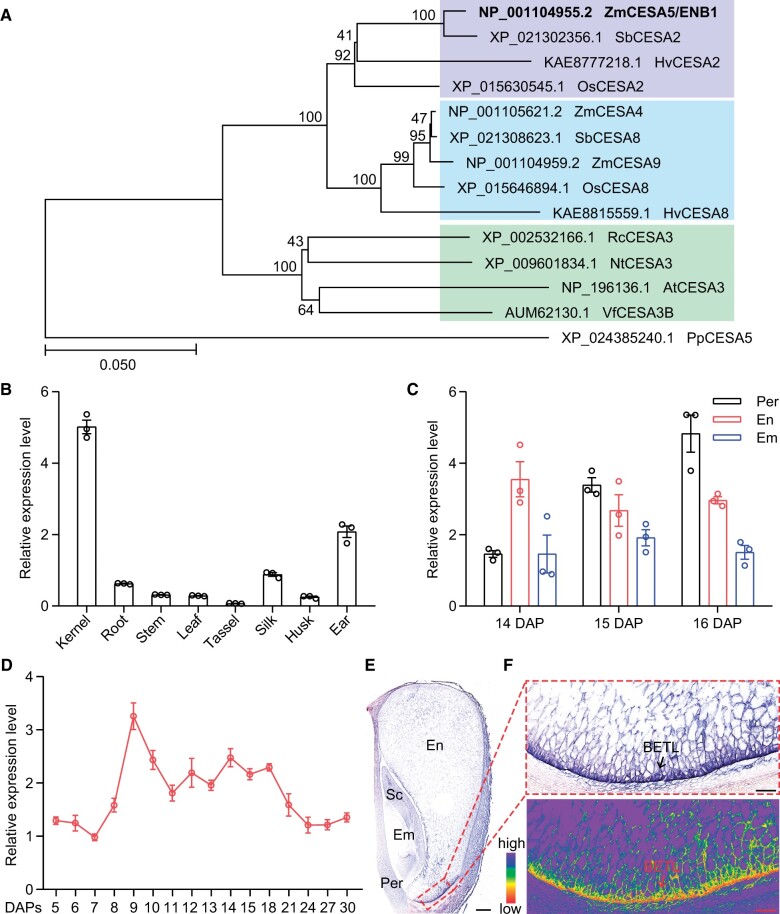
Phylogenetic relationships and expression pattern of ENB1. A, Phylogenetic relationships of ZmCESA5/ENB1 and homologs from other species. Zm, *Z. mays*; Sb, *Sorghum bicolor*; Os, *Oryza sativa Japonica Group*; Hv, *Hordeum vulgare*; Rc, *Ricinus communis*; Nt, *Nicotiana tabacum*; At, *A. thaliana*; Vf, *V. faba*; Pp, *Physcomitrella patens*. These sequences were aligned by the MUSCLE method, and the phylogenetic tree was constructed using the neighbor-joining method in the MEGA-X software package. The *P. patens* homologous protein was used as an outgroup. The purple and blue shadings represent ENB1 homologs in cereals, and the green shading represents ENB1 homologs in dicots. The ENB1 homologs of cereals are further divided into two sub-clades: one contains ENB1, another contains ZmCESA4 and ZmCESA9, which are represented with the purple and blue shadings, respectively. The numbers at the nodes represent the percentage of 1,000 bootstraps. Scale bar, the average number of AA substitutions per site. B–D, RNA expression pattern of *ENB1* in the various tissues (B), the three-component tissues of kernels (C), and the developing endosperms (D). In (B), Root, stem, leaf, tassel, silk, husk, and ear tissues were collected from W22 plants at the V12 stage. In (C), Per, En, and Em were isolated from 14 to 16 DAP W22 kernels. In (D), the endosperms were isolated from 5 to 30 DAP kernels. Data are mean ± sem (*n *= 3 biologically independent samples). E and F, mRNA ISH of *ENB1* using the 15 DAP W22 kernels. The upper panel of (F) is the magnified view of the region marked by the dashed box in (E). In (F), the lower panel is a pseudo-color image according to the gray value of the upper part (the lower the gray value, the more intense the ISH staining). Bars = 500 μm in (E), 100 μm in (F).

The DNA sequence of *ENB1* is 6,009-bp long, comprising 14 exons and 13 introns (Figure 2B). It encodes ∼120-kDa protein of 1,076 aa with two domains: a RING domain (18–63 aa) and a cellulose-synthase domain (352–1,071 aa; Figure 2C). Homology analysis showed that ENB1 shared significant sequence similarity with plant CESAs. CESAs constitute a protein family, and ENB1 is the homolog of CESA5 and has two closely related paralogs in maize, ZmCESA4 and ZmCESA9 (Supplemental Figure S3A; Supplemental Files S1 and S2). However, *ENB1* (*ZmCESA5*) exhibits a higher expression level than that of *ZmCESA4 and ZmCESA9* throughout kernel development (Supplemental Figure S3B). A phylogenetic tree indicated ENB1 homologs in cereals are specialized as a distinct clade (Figure 3A; Supplemental Files S3 and S4). Moreover, it divides them into two sub-clades: one contains ENB1, another contains ZmCESA4 and ZmCESA9 (Figure 3A).

Reverse transcription-quantitative PCR (RT-qPCR) analysis indicated *ENB1* was highly expressed in the kernel (Figure 3B). We examined *ENB1* expression in the three tissues (pericarp, endosperm, and embryo) of developing kernel and found *ENB1* was expressed in all of them, with higher expression in the endosperm and pericarp than the embryo (Figure 3C). During endosperm development, *ENB1* was expressed at all the stages tested (5–30 DAP) (Figure 3D). To investigate the site of *ENB1* action in endosperm, we examined its spatial expression by mRNA in situ hybridization (ISH) using 15 DAP W22 kernels. A remarkably strong hybridization signal was detected in BETL cells, while weak signals were detected in other endosperm cells (Figure 3, E and F; Supplemental Figure S4).

**Figure 4:**
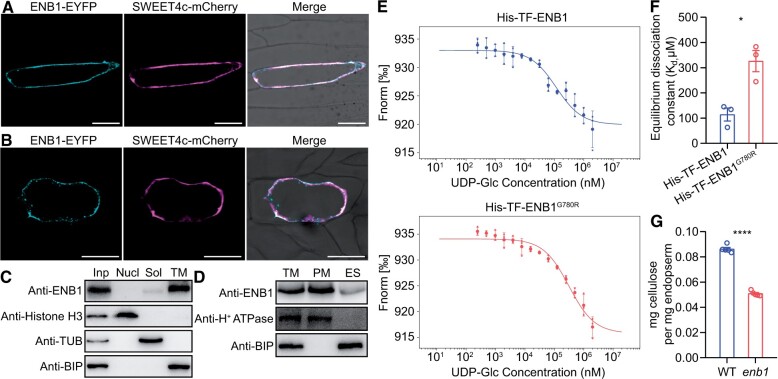
ENB1 shares the features of a plant CESA. A and B, Subcellular localization of ENB1 in normal (A) and plasmolytic (B) onion epidermal cells. The fluorescence signals of ENB1-EYFP and SWEET4c-mCherry were visualized using confocal laser scanning microscopy. Bar = 100 μm. C and D, Subcellular localization of ENB1 using the fractions isolated from 15 DAP W22 kernels. All fractions were examined using the corresponding marker antibodies to confirm the success of fractionation. Inp, input; Nucl, nuclear; Sol, soluble; TM, total membrane. E and F, Substrate binding assay of ENB1 and ENB^G780R^ (enb1) to substrate UDP-Glc. E, Microscale thermophoresis curves of ENB1 and ENB^G780R^ (enb1) binding to UDP-Glc. Fourteen dilutions of UDP-Glc were used, and the curves were fitted using the data of three independent experiments. F, Equilibrium *K_d_* of ENB1 and ENB^G780R^ (enb1) binding to UDP-Glc. Data are mean ± sem (*n *= 3 biologically independent samples). *P < 0.05; Student’s *t* test. G, Amount of cellulose in 12 DAP WT and *enb1* endosperms on a weight base. Data are mean ± sem (*n* = 5 biologically independent samples). ****P < 0.0001; Student’s *t* test.

The CESAs in plants assemble into the CSC and synthesize cellulose at the PM (McFarlane et al., 2014; Polko and Kieber, 2019). To examine the subcellular localization of ENB1, we transiently co-expressed ENB1-EYFP and a PM marker (SWEET4c-mCherry) in onion (*Allium cepa*) epidermal cells. The two signals highly overlapped (Figure 4, A and B). Tissue fractionation and immunoblotting analysis of 15 DAP W22 kernels showed ENB1 signals were strongly detected in the PM fraction, but they were also partially present in the endomembrane system (ES) and soluble fractions (Figure 4, C and D), which indicated that ENB1 was primarily located in the PM. Additionally, we observed ENB1-EYFP (SWEET4c-EYFP as a control) signals by spinning disk confocal microscopy, and found the ENB1-EYFP particles were mobile, while the signals of SWEET4c-EYFP were static (Supplemental Movies S1 and S2), which suggested ENB1 forms a CSC.

Next, using a microscale thermophoresis assay (Wienken et al., 2010), we examined whether ENB1 binds the substrate of CESAs, UDP-Glucose (UDP-Glc; Olek et al., 2014). A titration series from 244- to 2 mM UDP-Glc was conducted, with 100-nM catalytic domain (CatD) of ENB1 kept constant throughout the series. The detected interaction signals of ENB1 CatD and UDP-Glc indicated ENB1 CatD could directly bind UDP-Glc (Figure 4E; Supplemental Figure S5). We then examined whether the G780R mutation in ENB1 (ENB1^G780R^) affects UDP-Glc binding. Serial concentrations of UDP-Glc were titrated against 100-nM of ENB1^G780R^ CatD, and the interaction signals of the ENB1^G780R^ CatD and UDP-Glc were detected (Figure 4E; Supplemental Figure S5). Interestingly, the ENB1^G780R^ CatD increased the equilibrium dissociation constant (*K_d_*) with UDP-Glc (Figure 4F), indicating the G780R mutation decreased the binding capacity of ENB1 for UDP-Glc. A significantly decreased amount of cellulose in *enb1* endosperms further confirmed ENB1 is a CESA in maize (Figure 4G).

### *enb1* impairs flange ingrowth development and BETL function

**Figure 5:**
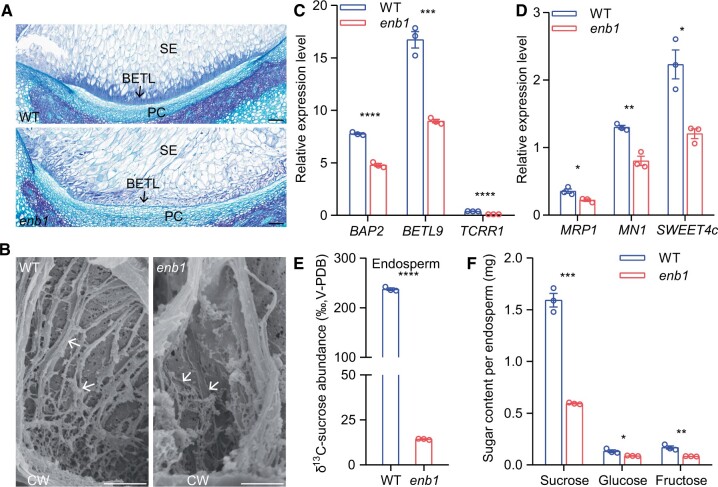
*enb1* impairs the development of flange ingrowths and the function of BETL cells. A, WT and *enb1* BETL cell observations at 15 DAP. Paraffin sections were stained by toluidine blue. PC, placento-chalazal region. Bar = 100 μm. B, High magnification observations of scanning electron microscopy showing ingrowths of BETL cells at 15 DAP in WT and *enb1* endosperms. The arrows indicate ingrowths. Bar = 5 μm. C and D, Expression of BETL-specific (C) and functionally-related (D) genes in 15 DAP WT and *enb1* endosperms. Data are mean ± sem (*n* = 3 biologically independent samples). ****P < 0.0001; ***P < 0.001; **P < 0.01; *P < 0.05; Student’s *t* test. E, ^13^C-sucrose abundance of 15 DAP WT and *enb1* endosperms. Data are mean ± sem (*n* = 3 biologically independent samples). ****P < 0.0001; Student’s *t* test. F, Sugar content of 15 DAP WT and *enb1* endosperms. Data are mean ±sem (*n* = 3 biologically independent samples). ***P < 0.001; **P < 0.01; *P < 0.05; Student’s *t* test.

Because *ENB1* was predominantly expressed in BETL cells, we examined these cells in paraffin sections stained with toluidine blue. The BETL cells of WT were filled with dense flange ingrowths, while those of *enb1* exhibited many fewer flange ingrowths (Figure 5A). We further observed the flange ingrowths by scanning electron microscopy and found they were thicker and more extensive in WT, and much thinner and disorganized in *enb1* (Figure 5B). These results indicated that *enb1* impaired the development of flange ingrowths in BETL cells.

To examine whether *enb1* impairs the development of BETL cells, we examined the expression levels of several BETL-specific genes, including *Basal Layer Antifungal Protein2* (*BAP2*), *BETL9*, and *Transfer Cell Response Regulator1* (*TCRR1*) by RT-qPCR. The decreased expression levels of these genes are consistent with a defective BETL cell phenotype in *enb1* (Figure 5C).

Next, we examined whether *enb1* affects the function of BETL cells. The downregulated expression levels of *MN1* and *SWEET4c* suggested impaired function of BETL cells in *enb1* (Figure 5D). Consequently, we tested the capacity for sucrose transport in BETL cells by a pulse-labeling assay using ^13^C-sucrose. We fed 15 DAP WT and *enb1* kernels with a medium containing ^13^C-sucrose for 12 h. Endosperms were isolated and ^13^C-sucrose abundance was measured by isotope ratio mass spectrometry. Compared with WT, the ^13^C-sucrose abundance in *enb1* endosperms was greatly decreased (Figure 5E). We measured the contents of three major sugars (sucrose, glucose, and fructose) in the endosperm by ion chromatography. Sugar accumulation of *enb1* endosperms was significantly reduced compared with WT (Figure 5F). Taken together, these results indicated *enb1* impaired the function of BETL cells.

### Impaired BETL function triggers endosperm breakdown

Next, we conducted transcriptome profiling to assess the effects of impaired BETL function on gene expression in WT and *enb1* endosperms at 15 DAP. A total of 5,389 significant differentially expressed genes (DEGs) were identified (Supplemental Data Set 1). The downregulated expression of what are typically plentiful BETL-predominately expressed DEGs was consistent with defective BETL cells in *enb1* (Supplemental Figure S6).

Gene ontology (GO) analysis showed downregulated DEGs correspond to four major GO terms (Supplemental Figure S7A; Supplemental Data Set 2). In maize endosperm, starch synthesis occurs in the amyloplast, a specialized plastid. The DEGs of “plastid organization” and “starch synthesis,” including *Brittle endosperm2* and *Shrunken2*, were downregulated in *enb1* (Supplemental Figure S7B). By transmission electron microscopy (TEM), we observed fewer and smaller starch granules (SGs) in *enb1* (Supplemental Figure S7, C–E). Starch is synthesized in the endosperm from sucrose transported from maternal tissue via BETL cells; therefore, disrupted starch synthesis is consistent with the reduced sucrose transport by BETL cells in *enb1*. Although protein synthesis was also affected, the expression of genes encoding zein storage proteins was not significantly altered in *enb1* (Supplemental Data Set 2).

**Figure 6:**
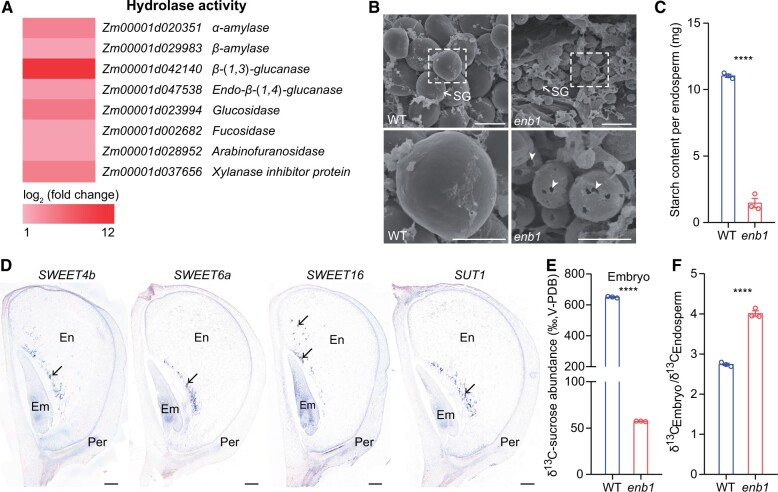
Sugar of DE starch is transported to the embryo in *enb1*. A, Heatmap depicting the log_2_ (fold change) of representative DEGs of hydrolase activity in 15 DAP WT and *enb1* endosperms. B, Scanning electron microscopy observations of SGs of 15 DAP WT and *enb1* endosperms. The lower panels are the magnified sections from the white dashed boxes of the upper panels. White arrowheads indicate distinct surface pitting of SGs. Bars = 10 μm (upper), 5 μm (lower). C, Starch amount of 15 DAP WT and *enb1* endosperms. Data are mean ± sem(*n* = 3 biologically independent samples). ****P < 0.01; Student’s *t* test. D, mRNA ISH of *SWEET*s and *SUT1* (upregulated DEGs) using the 15 DAP W22 kernels. Bar = 500 μm. E, ^13^C-sucrose abundance of 15 DAP WT and *enb1* embryos. Data are mean ±  sem (*n *= 3 biologically independent samples). ****P < 0.0001; Student’s *t* test. F, Ratio of ^13^C-sucrose abundance of embryos to endosperms in 15 DAP WT and *enb1* kernels . Data are mean ±  sem (*n* = 3 biologically independent samples). ****P < 0.0001; Student’s *t* test.

GO analysis showed that upregulated DEGs in *enb1* correspond to six major terms (Supplemental Figure S8A; Supplemental Data Set 3). Most of the DEGs of “hydrolase activity, acting on glycosyl bonds” were starch and CW hydrolysis-encoding genes (Figure 6A; Supplemental Data Set 3). To examine whether starch was being degraded, we observed SGs by scanning electron microscopy. At 15 DAP, SGs of *enb1* exhibited distinct surface pitting, with some manifesting hollow shells due to loss of internal material, while those of WT were intact with smooth surfaces (Figure 6B). The SGs of *enb1* resembled starch grains subjected to amylase digestion (Dhital et al., 2014). Thus, the drastically reduced starch content of endosperm in *enb1* was consistent with severe degradation and reduced synthesis of starch (Figure 6C).

Intriguingly, the GO term “transporter activity” included sugar transporter-encoding genes, such as *Sugar Will Eventually be Exported Transporter* (*SWEET*) and *Sucrose Transporter1* (*SUT1*) (Supplemental Data Set 3). We randomly selected three *SWEET*s and *SUT1* to examine their spatial expression by mRNA ISH using 15 DAP W22 kernels. The hybridization signals of these DEGs were strongly detected flanking the embryo/endosperm interface (Figure 6D; Supplemental Figure S8B), indicating that the capacity for sugar transport in this region was enhanced in *enb1*. We examined the potential for sucrose transport in *enb1* embryos using the ^13^C-sucrose-labeled kernels for which ^13^C-sucrose abundance in their endosperms was previously measured (Figure 5E). The result indicated a greatly decreased ^13^C-sucrose abundance in the *enb1* embryo compared with WT (Figure 6E). Intriguingly, we found higher ^13^C-sucrose abundance by the embryo than the endosperm in both the WT and *enb1* (Figures 5, E and 6, E), suggesting the sink strength of the embryo was greater than that of the endosperm. Moreover, the ratio of ^13^C-sucrose abundance of the *enb1* embryo to endosperm was significantly higher than that of WT (Figure 6F).

Genes corresponding to “oxidoreductase activity,” “cofactor binding,” “tetrapyrrole binding,” and “ion binding” are involved in important redox processes, particularly in the production and scavenging of reactive oxygen species (ROS; Supplemental Figure S8C; Supplemental Data Set 3), and ROS can induce programmed cell death (PCD) in plants (Young and Gallie, 2000; Cheng et al., 2016). We examined PCD in the endosperm by cell viability staining with Evans blue. During maize kernel development, the SE cells undergo a particular nonlytic PCD process beginning around 16 DAP (Young and Gallie, 2000). Compared with WT, developing endosperms of *enb1* exhibited deeper and more extensive staining, especially at 18 DAP, indicating endosperm cells of *enb1* manifested more widespread cell death (Supplemental Figure S8D).

### Impaired BETL function leads to upregulated expression of genes involved in hormone response and carbohydrate and lipid metabolism in the embryo

**Figure 7:**
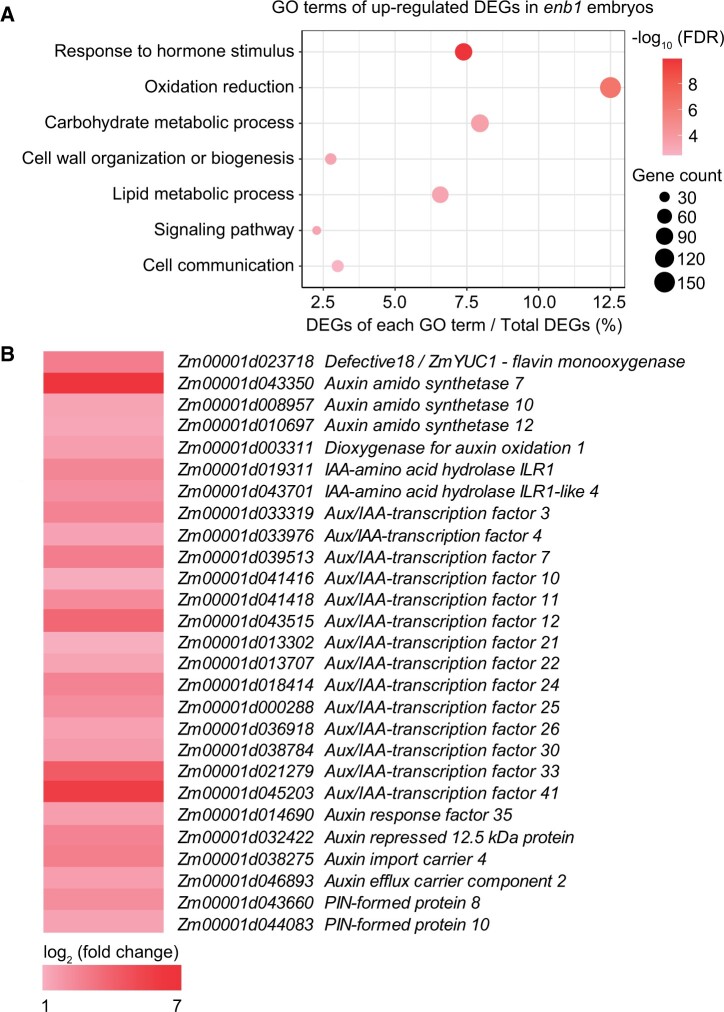
*enb1* upregulates the expression of genes involved in hormone response, and carbohydrate and lipid metabolism of the embryo. A, GO analysis of upregulated DEGs in *enb1* embryos. Circle sizes indicate DEG numbers and the color gradients indicate enrichment significance. B, Heatmap depicting the log_2_ (fold change) of representative upregulated auxin-related genes in *enb1* embryos.

We conducted transcriptome profiling to investigate gene expression in *enb1* embryos at 15 DAP, when its endosperm transitioned from a period of growth to a period of degradation. A total of 2,147 DEGs were identified, including 554 and 1,593 that were downregulated and upregulated, respectively, in *enb1* embryos (Supplemental Data Set 4). GO enrichment analysis showed that the downregulated DEGs were not classified into specific GO terms, while upregulated DEGs were mainly classified into seven GO terms (Figure 7A). Genes corresponding to “response to hormone stimulus,” “oxidation reduction,” and “signaling pathway” are involved in the synthesis, metabolism, transport, and response to hormones, especially auxin (Figure 7B; Supplemental Data Set 5). Interestingly, *Defective18* (*DE18*)*/ZmYUC1*, encoding a rate-limiting enzyme in auxin synthesis (Bernardi et al., 2012; Zhao, 2018), was upregulated in *enb1* embryos but downregulated in *enb1* endosperms (Supplemental Data Sets 1 and 4). These results suggested that auxin plays important roles during endosperm breakdown and embryo growth in *enb1*. Additionally, GO terms associated with “carbohydrate metabolic process,” “cell wall organization or biogenesis,” and “lipid metabolic process” DEGs were also found to be enriched, which suggested enhanced metabolism in *enb1* embryos. Regarding the GO term “cell communication,” 15 of the 37 DEGs encode kinases and receptor-like kinases (Supplemental Data Set 5). The expression levels of *Mitogen-Activated Protein Kinase 1* (*MAPK1*), *MAPK3*, *MAPKK2*, and *SNF1-related protein kinase β1* were substantially increased in *enb1* embryos (Supplemental Data Set 5), suggesting enhanced kinase-mediated cellular signaling.

### Low sucrose supply induces endosperm breakdown

Defective BETL cells resulted in drastically reduced sucrose transport from the maternal plant to the endosperm, and ultimately this led to endosperm breakdown in *enb1*. To investigate the effect of sucrose supply on endosperm development, we cultured 4 DAP W22 kernels for 10 and 20 days in vitro in a medium containing either 15% sucrose (high sucrose) or 3% sucrose (low sucrose; Gruis et al., 2006; Olsen, 2020). In high sucrose, the size of kernels increased from 10 to 20 days, while kernel size was not notably increased in low sucrose medium during the same period.

**Figure 8:**
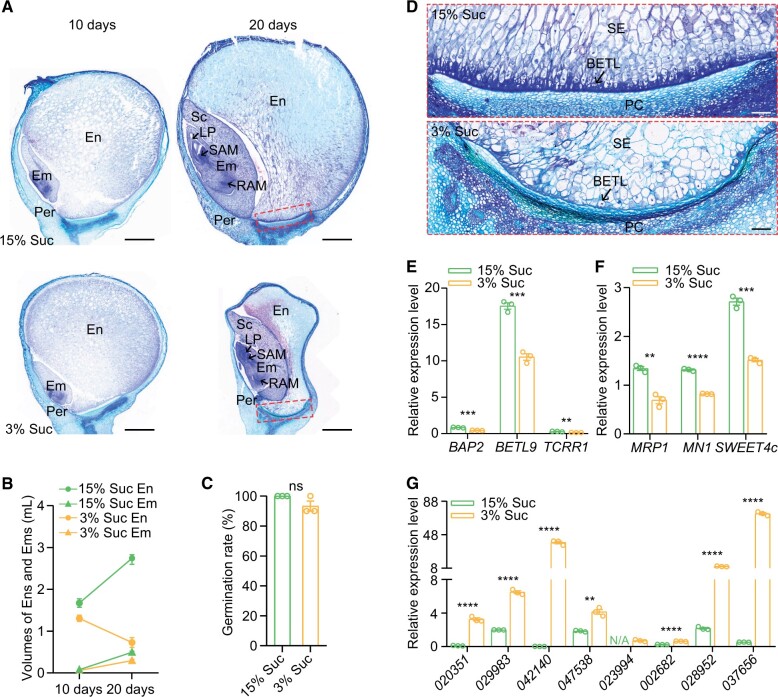
Low sucrose supply induces endosperm breakdown. A, Paraffin section observations of developing kernels cultured with different sucrose levels. Per, pericarp; En, endosperm; Em, embryo; Sc, scutellum; LP, leaf primordia; SAM, shoot apical meristem; RAM, root apical meristem. Bar = 1 mm. B, Volumes of 30 endosperms (Ens) and 30 embryos (Ems) of kernels cultured in 15% or 3% sucrose. Data are mean ± sem (*n* = 3 biologically independent samples). C, Germination rate of kernels cultured for 20 days in 15% or 3% sucrose. Data are mean ± sem (*n* = 3 biologically independent samples); ns, not significant; Student’s *t* test. D, BETL cell observations of kernels cultured for 20 days in 15% or 3% sucrose. The panels are magnifications of the regions marked in red dashed boxes in (A). Bar = 100 μm. E and F, Expression of BETL-specific expressed (E) and functionally related (F) genes of endosperms, which were isolated from kernels cultured for 20 days in 15% or 3% sucrose. Data are mean ± sem (*n* = 3 biologically independent samples). ****P < 0.0001; ***P < 0.001; **P < 0.01; Student’s *t* test. G, Expression of genes involved in hydrolase activity in the endosperms that were isolated from kernels cultured for 20 days in 15% or 3% sucrose. NA, not applicable. The numbers represent the gene ID, for example, “*020351*” represents “*Zm00001d020351*.” Data are mean ± sem (*n* = 3 biologically independent samples). ****P < 0.0001; ***P < 0.001; **P < 0.01; Student’s *t* test.

We paraffin-sectioned these kernels to observe development of the endosperm and embryo. In high sucrose, the endosperms and embryos enlarged from 10 to 20 days (Figure 8, A and B). Intriguingly, in low sucrose, the embryos enlarged, while the endosperms became substantially depleted during the same period (Figure 8, A and B), indicating the endosperms were becoming degraded. Moreover, embryos in low sucrose formed typical embryonic structures (Figure 8A). We isolated embryos from the cultured kernels at 20 days and found that their germination rate showed no significant difference between the high and low sucrose (Figure 8C). These results indicated that the low sucrose supply caused degradation of the endosperm but sustained development of a fully viable embryo.

To investigate whether development of WT kernels cultured in low sucrose was similar to what occurs in *enb1*, we examined several key biological processes. We observed severely decreased BETL CWIs in low sucrose (Figure 8D). Moreover, they showed reduced expression of BETL-specific and functionally related genes, indicating that the low sucrose supply suppressed the development and function of BETL cells (Figure 8, E and F). Like *enb1*, key starch synthetic genes, including *Brittle endosperm2* and *Shrunken2*, exhibited drastically reduced expression (Supplemental Figure S9A), suggesting that the low sucrose supply reduced starch synthesis. However, expression of genes involved in hydrolase (including amylase) activity was increased (Figure 8G), suggesting that the low sucrose supply triggered starch degradation. Additionally, *DE18* exhibited drastically decreased expression (Supplemental Figure S9B), suggesting that the low sucrose supply reduced auxin synthesis.

### Sucrose induces *ENB1* expression by MRP1

**Figure 9:**
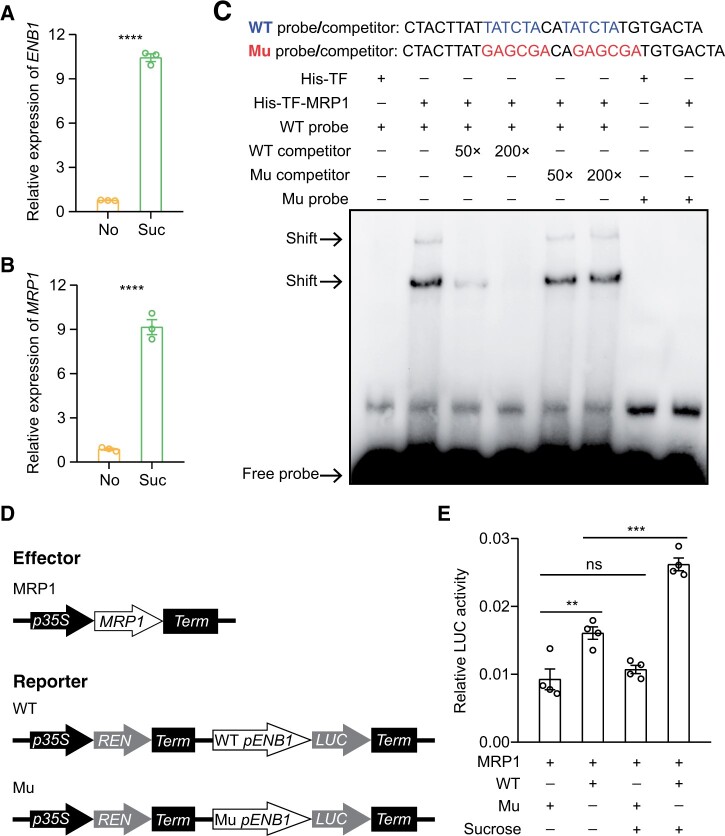
Sucrose induces *ENB1* expression by MRP1. A, Expression of *ENB1* in the endosperms cultured on mediums containing 3% sucrose or no sugar. Data are mean ± sem (*n* = 3 biologically independent samples). ****P < 0.0001; Student’s *t* test. B, Expression of *MRP1* in endosperms cultured on medium containing 3% sucrose or no sugar. Data are mean ± sem (*n* = 3 biologically independent samples). ****P < 0.0001; Student’s *t* test. C, EMSAs of the MRP1 and *ENB1* promoters. Competition assays for the biotin-labeled probe were conducted by adding 50, 200-fold excess of unlabeled WT or mutated probe. Mutation assays for the biotin-labeled probe were conducted by mutating the TATCTA motif. D, Schematic diagram shows constructs used in the dual-LUC transient transcriptional activity assays. E, Transcriptional activity of MRP1 on the *ENB1* promoter after sucrose treatment of maize protoplasts. Data are mean ± sem (*n* = 4 biologically independent samples). ***P < 0.001; **P < 0.01; ns, not significant; Student’s *t* test.

We established that *ENB1* expression can be induced by sucrose during in vitro kernel culture (Figure 9A). Additionally, *Myb-Related Protein1* (*MRP1*), encoding a key transcription factor involved in the differentiation and phenotypic maintenance of BETL cells (Gomez et al., 2009), is also induced by sucrose during in vitro kernel culture (Figure 9B). We therefore investigated whether *ENB1* expression is regulated by MRP1, and whether its regulation is dependent on sucrose signaling. The binding motif of MRP1 to target genes is thought to be TATCTA/C (Chourey and Hueros, 2017), so we tested whether MRP1 can bind this sequence in the *ENB1* promoter using an electrophoretic mobility shift assay (EMSA). DNA mobility shifts were detected by EMSA (Figure 9C). Band shifts were drastically weakened after adding excess WT competitor, while the weakened band shifts failed to occur after adding excess mutated competitor (Figure 9C). Moreover, no shifted bands were detected when the labeled probe contained mutated TATCTA motifs (Figure 9C). These results indicated that MRP1 can bind directly to the *ENB1* promoter in vitro.

We next conducted a dual-luciferase (LUC) transient transcriptional activity assay in maize leaf protoplasts. We used the 35S cauliflower Mosaic Virus (CaMV) promoter to drive expression of *MRP1*, the *REN* internal control, and the *ENB1* promoter containing a WT or mutated binding motif on the *LUC* reporter gene (Figure 9D). A significantly increased LUC activity was detected in protoplasts when MRP1 was co-expressed with the reporter containing the WT binding motif (Figure 9E), indicating that MRP1 transactivated the *ENB1* promoter. We treated the transformed protoplasts with 1-mM sucrose, and this strongly enhanced the activity of the reporter harboring the WT binding motif, while it did not occur with the reporter harboring the mutated binding motif (Figure 9E). These results indicated that sucrose induced *ENB1* expression through MRP1.

### Overexpression of *ENB1* enhances BETL function and increases kernel weight

**Figure 10:**
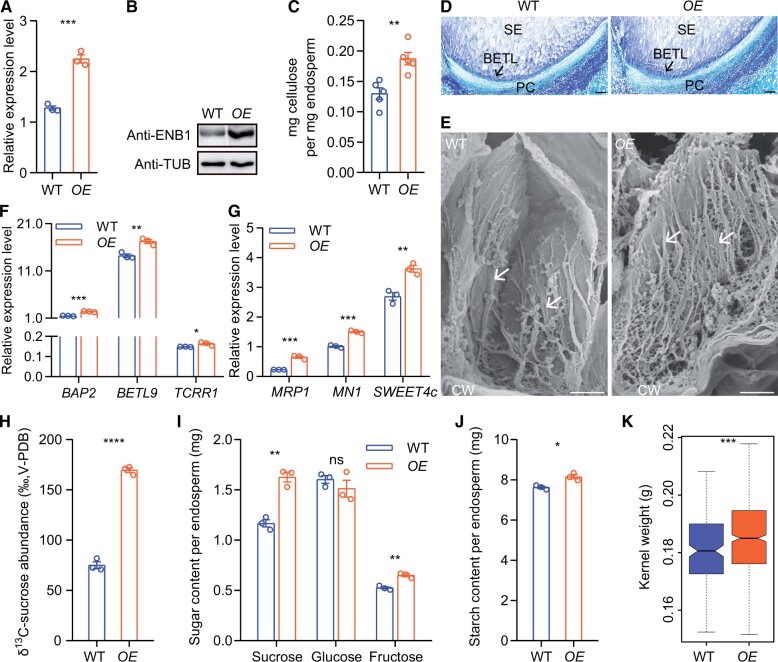
*ENB1* overexpression enhances the development of flange ingrowths and the function of BETL cells. A, Expression of *ENB1* in 12 DAP WT and *OE* endosperms. Data are mean ± sem (*n* = 3 biologically independent samples). ***P < 0.001; Student’s *t* test. B, Protein accumulation of ENB1 in 12 DAP WT and *OE* endosperms. C, Amount of cellulose in 12 DAP WT and *OE* endosperms on a weight base. Data are mean ± sem (*n* = 5 biologically independent samples). **P < 0.01; Student’s *t* test. D, WT and *OE* BETL cell observations at 12 DAP. Paraffin sections were stained with toluidine blue. Bar = 100 μm. E, High magnification observations of scanning electron microscopy showing ingrowths of BETL cells at 12 DAP in WT and *OE* endosperms. The arrows indicate ingrowths. Bar = 5 μm. F and G, Expression of BETL-specific (F) and functionally related (G) genes in 12 DAP WT and *OE* endosperms. Data are mean ± sem (*n* = 3 biologically independent samples). ***P < 0.001; **P < 0.01; *P < 0.05; Student’s *t* test. H, ^13^C-sucrose abundance of 12 DAP WT and *OE* endosperms. Data are mean ± sem (*n* = 3 biologically independent samples). ****P < 0.0001; Student’s *t* test. I, Sugar content of 12 DAP WT and *OE* endosperms. Data are mean ±sem (*n* = 3 biologically independent samples). **P < 0.01; ns, not significant; Student’s *t* test. J, Starch content of 12 DAP WT and *OE* endosperms. Data are mean ± sem (*n* = 3 biologically independent samples). *P < 0.05; Student’s *t* test. K, Kernel weight of WT and *OE* mature kernels that were from two transgenic lines (two ears per line). *n* = 193 kernels in WT, *n* = 636 kernels in *OE*. ***P < 0.001; Wilcoxon rank sum test.

We overexpressed *ENB1* driven by its native promoter (*ProENB1:ENB1*, *OE*) to investigate the effects of elevated expression of *ENB1* on endosperm development. *ENB1* expression in *OE* endosperms was elevated both in RNA and protein levels compared with WT (Figure 10, A and B). Moreover, the increased cellulose content in *OE* endosperms confirmed successful overexpression of *ENB1* (Figure 10C). We observed the BETL cells and found they exhibited deeper staining with toluidine blue in *OE* endosperm compared with WT (Figure 10D). Furthermore, scanning electron microscopy observations revealed denser flange ingrowths in *OE*, indicating overexpression of *ENB1* enhanced development of flange ingrowths (Figure 10E).

The elevated expression of BETL-specific and functionally related genes suggested that overexpression of *ENB1* enhanced the development and functional activity of BETL cells in *OE* (Figure 10, F and G). Consequently, we tested the capacity for sucrose transport in BETL cells by culturing 12 DAP WT and *OE* kernels in a medium containing ^13^C-sucrose for 12 h. Compared with WT, the ^13^C-sucrose abundance of *OE* endosperms was increased (Figure 10H). In addition, sugar and starch contents in the endosperms were greater, indicating that they were more abundant in *OE* endosperms compared with WT (Figure 10, I and J). Together, these results demonstrate that *ENB1* overexpression enhances the functional activity of BETL cells.

We examined the developmental status of endosperm cells by measuring the degree of endoreduplication. Flow cytometry indicated that endoreduplicated nuclei of 12C and above accounted for 28.37% of the DNA in *OE*, but 26.34% of the DNA in WT, which was due to significantly increased DNA content of 48C and ≥96C in *OE* compared with WT (Supplemental Figure S10), suggesting that the *OE* endosperm had elevated metabolic activity. We also measured the kernel weight of mature kernels and found that *OE* kernels were 2.15% heavier than those of the WT (Figure 10K).

## Discussion

Without direct evidence, it has been postulated CWIs increase the BETL cell PM surface area, enhancing sugar transport into the endosperm. Our characterization of ENB1 and the *enb1* mutant provides clear evidence that the extent of wall ingrowth formation directly affects nutrient update in BETL cells; furthermore, it shows that a specialized CESA/CSC is involved in ingrowth formation. CESAs are a protein family in plants, and their number varies among species (Popper et al., 2011). There are 10 CESAs in the Arabidopsis genome, but in maize 18 CESAs are annotated according to the B73 reference genome (maize B73 RefGen_V4) (Jiao et al., 2017; Penning et al., 2019). CESAs in Arabidopsis have been extensively studied, but those in maize have not been functionally characterized. We identified ENB1 as ZmCESA5, a member of maize CESAs. Our results indicated that ENB1 is a PM protein and can bind the substrate of CESAs, UDP-Glc (Figure 4, A–F). Moreover, the *enb1* mutation (G780R mutation in ENB1) impairs binding of UDP-Glc and reduces cellulose content in *enb1* endosperm (Figure 4, F and G). This mutation is three amino acids away from the third D of the conserved D, D, D, and QXXRW residues that are critical for substrate binding and catalysis of plant CESAs (Fujita et al., 2013; McFarlane et al., 2014). In Arabidopsis, mutations that are at or close to the third D (e.g. D780N and E779K in AtCESA1, and D683N and S679L in AtCESA8) in CESAs also lead to a dramatic reduction in cellulose synthesis (Taylor et al., 2000; Beeckman et al., 2002). Therefore, ENB1 as a key functional CESA protein in maize shares several common features of plant CESAs.

Our study revealed that ENB1 is responsible for directing the synthesis of flange ingrowths in BETL cells of maize endosperm (Figures 5, A and B and 10, D and E). However*, ENB1* is also expressed in other tissues including pericarp, where there are no flange ingrowths. Plant CESAs are generally assembled into CSCs, where each CSC is a trimer with three different CESAs synthesizing cellulose deposited into CWs (Gonneau et al., 2014; Hill et al., 2014; McFarlane et al., 2014). In Arabidopsis, AtCESA1, AtCESA3, and AtCESA6-like are involved in primary CW synthesis, while AtCESA4, AtCESA7, and AtCESA8 are required for secondary CW synthesis (McFarlane et al., 2014; Polko and Kieber, 2019). The mobility of ENB1-EYFP particles suggested that ENB1 forms a CSC. The phylogenetic tree shows ENB1 has two closely related paralogs in maize, ZmCESA4 and ZmCESA9, but it exhibits a higher expression level than those two paralogs. It is possible that functionally different CESA combinations arise from different CSCs and synthesize different types of celluloses in different tissues. We speculate that ENB1 forms a CSC, probably with ZmCESA4 and ZmCESA9, for synthesizing flange ingrowths in BETL cells, and it forms CSCs with other CESAs for synthesizing different celluloses in other tissues.

**Figure 11:**
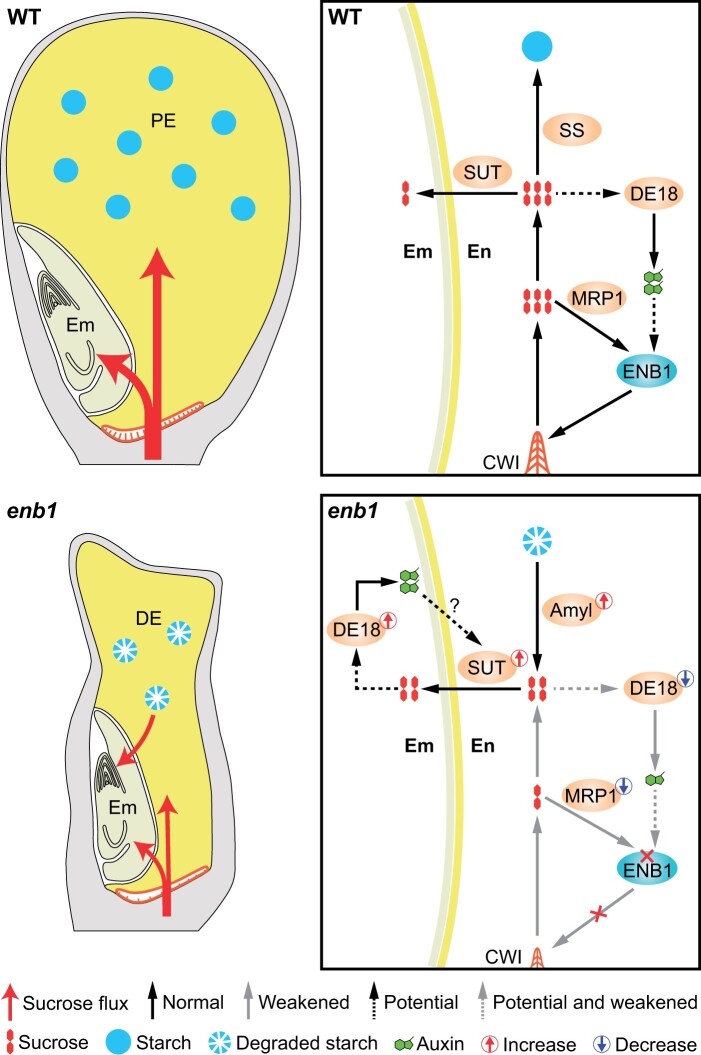
Model illustrating that CWIs synthesized by ENB1 enhance sucrose supply to sustain development of a PE. In WT, ENB1 in BETL cells directs synthesis of CWIs that transport sucrose and further induces *ENB1* through MRP1. Additionally, the sucrose–auxin signaling axis also contributes to CWI development through *ENB1*. Ultimately, ENB1 mediates the formation of well-developed CWIs that enhance sucrose import. The sucrose is then transported to endosperm cells that synthesize and accumulate storage metabolites, such as starch, while also nourishing the embryo. The *enb1* mutation impairs CWI formation, which severely reduces sucrose transport, thereby resulting in suppressed starch accumulation in the endosperm. The enhanced auxin signaling in the *enb1* embryo may act at the embryo/endosperm interface, upregulating the expression of genes encoding to enhance the sucrose supply from the endosperm to the growing embryo. The *enb1* embryo with high sink strength triggers endosperm starch degradation, resulting in endosperm breakdown. SS, starch synthase; Amyl, amylase.

The results of experiments using maize kernel culture indicate sucrose induces the formation of flange ingrowths in BETL cells, which promote the transport of high levels of nutrients, including sucrose (Pate and Gunning, 1972; Felker and Shannon, 1980; Offler and Patrick, 1993; Offler et al., 2003; Chourey and Hueros, 2017). It has been proposed that the cereal endosperm could have evolved under a high sucrose content (Olsen, 2020). In vitro maize endosperm organ culture showed high sucrose (15% sucrose) influenced cell fate specification of the aleurone and SE, but not the BETL cells; low sucrose (3% sucrose) resulted in a large proportion of nondifferentiated endosperm cells that lacked internal organization (Gruis et al., 2006; Olsen, 2020). Interestingly, we found that sucrose induces *ENB1* expression through MRP1 (Figure 9), and a mutation of *ENB1* impairs the formation of flange ingrowths in BETL cells (Figure 5, A and B). Consequently, sucrose induces *ENB1* expression, promoting the synthesis of flange ingrowths, which transport more sucrose to induce *ENB1* expression. This “feed-forward” cycle ultimately establishes highly developed flange ingrowths in BETL cells, contributing to grain filling (Figure 11). The ENB1-mediated reinforced regulation between sucrose and flange ingrowth development resembles that between sugar and BETL cell development via SWEET4c (Sosso et al., 2015). Therefore, sucrose is not only a nutrient, but also an important signaling molecule affecting the differentiation and development of endosperm cells.

In maize, a mutation of *DE18*, which encodes an auxin synthesis rate-limiting enzyme, greatly decreases free IAA content in endosperm to <10% compared with WT (Bernardi et al., 2012), and impairs the development of flange ingrowths in BETL cells (Forestan et al., 2010). In broad bean (*Vicia faba*), auxin induces the deposition of reticulate ingrowths, making adaxial epidermal cells of cotyledons trans-differentiate into transfer cells (Dibley et al., 2009). These studies suggested that auxin is important for the development of CWIs. Our results indicate that the expression level of *DE18* is greatly affected by sucrose levels (Supplemental Data Set 1; Supplemental Figure S9B), suggesting that local auxin levels exhibit sucrose responsive changes in the endosperm. In the *enb1* endosperm, the low sucrose level suppressed the expression of *DE18*, which could lead to low auxin level in the endosperm. The low auxin signaling could further suppress the development of flange ingrowths in BETL cells. Interestingly, promoter analysis using PlantPAN3.0 (http://plantpan.itps.ncku.edu.tw/) predicts that the promoter of *ENB1* contains auxin responsive *cis*-elements (“TGTCGG” and “TGTCTC”) for auxin response factors (ARFs; GalLi et al., 2018; Liang et al., 2020), suggesting that *ENB1* could be regulated by ARFs through auxin signaling. We thus speculate that such a sucrose–auxin signaling axis could regulate flange ingrowth development in BETL cells through *ENB1* (Figure 11).

The dramatically reduced level of maternal sucrose import in *enb1* results in a DE, but a fully viable embryo. We investigated whether the endosperm breakdown in *enb1* was due to the greater sink strength from the embryo. Our results indicate enhanced sucrose transport to the embryo (Figure 6, D and F). The increased sugar level might enhance the auxin level in the *enb1* embryo through the sucrose-responsive *DE18*. Indeed, multiple auxin-related genes are upregulated in the *enb1* embryo, suggesting that the *enb1* embryo has an increased local auxin level and enhanced auxin signaling. The endosperm sub-domain (embryo/endosperm interface) adjacent to the scutellum (Sc) of the embryo is enriched with putative sugar transporters (Doll et al., 2020). Interestingly, our results indicate that sugar transporters specifically expressed in this endosperm sub-domain are strongly upregulated in the *enb1* endosperm. Promoter analysis using PlantPAN3.0 predicts that the promoters of these sugar transporter encoding genes also include the binding sites (“TGTCGG” or “TGTCTC”) for ARFs, suggesting that they could be regulated by auxin. Therefore, we speculate that enhanced auxin signaling from the *enb1* embryo may act at the embryo/endosperm interface, upregulating the expression of sugar transporters to enhance the sugar supply from the endosperm to the growing embryo (Figure 11). Indeed, in rose (*Rosa hybrid*), auxin also regulates sucrose transport through a RhARF7–*RhSUC2* module in the abscission zone (Liang et al., 2020).

In summary, the flange ingrowths of BETL cells are synthesized by a CSC containing ENB1 (Figure 11). The ingrowths allow high levels of sugar transport from the maternal plant to the kernel, facilitating the development of endosperm cells while providing the sugar needed to meet the higher sink strength of the embryo. A persistent endosperm (PE) results from acquiring carbohydrates and other nutrients in excess of the needs of the embryo, making the endosperm a storage organ in cereal kernels. *ENB1* appears to be necessary for the unusual pattern of cellulose synthesis in BETL cells that is responsible for their development and function. *ENB1* overexpression enhanced BETL function and increased kernel weight, suggesting that *ENB1* is a target for engineering yield improvement in cereal crops.

## Materials and methods

### Plant materials

The maize (*Z.* *mays*) *5512k* (*enb1*) mutant was obtained from the Maize Genetics Cooperation Stock Center. *enb1* was crossed into the W22 genetic background, and kernels were harvested from self-pollinated +/*enb1* ears. Root, stem, the third leaf, tassel, silk, husk, and ear tissues were harvested from at least three W22 plants at the V12 stage as previously described (Wang et al., 2012). Developing kernels were harvested from W22 ears from 5 to 30 DAP. All plants were cultivated in the field or the greenhouse at the campuses of China Agricultural University (Beijing, China) or Shanghai University (Shanghai, China).

### Cytological observations

Developing kernels were freshly collected and prepared for paraffin sectioning. Briefly, these kernels were fixed with 3.7% FAA (3.7% (v/v) formalin, 50% (v/v) ethanol, and 5% (v/v) acetic acid), dehydrated with gradient ethanol (50%, 70%, 85%, 95%, and 100% ethanol in H_2_O [v/v]), substituted with xylene, and eventually embedded in Paraplast Plus (Sigma-Aldrich, St. Louis, MO, USA; cat. no. P3683). The 8-μm sections were stained with 0.1% (m/v) toluidine blue (Sigma-Aldrich, St. Louis, MO, USA; cat. no. 89640) and imaged with the digital microscope Pannoramic MIDI (3D HISTECH, Budapest, Hungary).

For TEM analysis, developing kernels were freshly collected and treated as described previously (Qi et al., 2016). The sections were then observed using the H7600 (Hitachi, Tokyo, Japan).

For scanning electron microscopy analysis of starch grains, the developing kernels were freshly collected and treated according to a previously described protocol (Wang et al., 2011). The scanning electron microscopy analysis of BETL cells was performed as previously described (Talbot et al., 2002). These samples were then observed using the S-3400N (Hitachi) or TM4000 Plus (Hitachi, Tokyo, Japan).

### Positional cloning

Two equivalent genomic DNA pools extracted from 40 individual WT and *enb1* kernels, respectively, were prepared for the maizeSNP3072 genotyping array as described previously (Tian et al., 2015). The mutant locus was mapped to a 1.5-Mb region using the 108 F_2_  *enb1* individuals and ultimately placed in a 287.90-kb region with the markers AC529 and Indel3 using the 1,937 F_2_  *enb1* individuals. The eight predicted genes in the 287.90-kb region were amplified from the homozygous WT and *enb1* plants and sequenced for the DNA alignment analyses. Relevant primer sequences are given in Supplemental Data Set 6.

### Functional complementation test

A 9,948-bp genomic DNA fragment containing the entire *ENB1* gene, the 2,685-bp upstream region, and the 2,068-bp downstream region was isolated from the W22 inbred line. The fragment was inserted into the binary vector, pTF102, to generate the *ProENB1: ENB1* construct. The resulting plasmid was introduced into the Agrobacterium (*Agrobacterium tumefaciens*) strain EHA105, and maize transformation was performed using immature zygotic embryos of the Hi-II hybrid (pApB) as described previously (Frame et al., 2002). Seven independent transgenic lines were obtained, four of which were crossed to *enb1* heterozygous plants and then selfed to obtain F_2_ ears for a functional complementation test. Relevant primer sequences are given in Supplemental Data Set 6.

### Phylogenetic analysis

A Basic Local Alignment Tool for Protein search was done with the full-length ENB1 protein sequence to obtain the relevant homologs sequences of other species in the National Center for Biotechnology Information (NCBI) nonredundant protein sequences database. AA sequences were aligned by the MUSCLE method, and a phylogenetic tree was constructed by the neighbor-joining method in the MEGA-X software package. The evolutionary distances were computed using the Poisson correction analysis. The bootstrap method with 1,000 replicates was used for testing the reliability of the interior branches of the phylogenetic tree.

### mRNA ISH

mRNA ISH was performed as described previously with minor modifications (Zhang et al., 2018). The CESAs of higher plants include two variable regions (VR1 and VR2; Polko and Kieber, 2019), and the VR1 fragment of *ENB1* was used for designing the probe. For the *SWEET*s and *SUT1*, the fragments including the part 3′-UTR sequence were used for designing the probe. These antisense and sense probes were synthesized and labeled using the DIG RNA Labeling Kit (SP6/T7) (Roche, Basel, Switzerland; cat. no. 11175025910) according to the manufacturer’s instructions. The 15 DAP kernels of the W22 inbred line were collected and prepared for paraffin sectioning. The 8 μm sections were hybridized with the probes, and then reacted with Anti-Digoxigenin-AP (Roche, Basel, Switzerland; cat. no. 11093274910), and ultimately detected with NBT/BCIP (Roche, Basel, Switzerland; cat. no. 11681451001). Relevant primer sequences are given in Supplemental Data Set 6.

### Subcellular localization

The full-length open reading frame (ORF) of *ENB1* was cloned into the transient expression vector pSAT6-EYFP-N1. Transient expression assays in onion (*A.* *cepa*) were performed as described previously (Li et al., 2018). The fluorescence signals were observed and imaged with the confocal laser-scanning microscope LSM710 (Zeiss, Oberkochen, Germany). Relevant primer sequences are given in Supplemental Data Set 6.

The 15 DAP W22 kernels were freshly collected and quickly cut into small pieces (1–2 mm × 1–2 mm). Approximately a 300-mg sample was used for the fractionation with the Minute PM Protein Isolation Kit for Plants (Invent Biotechnologies, Plymouth, MN, USA; cat. no. SM-005-P) according to the manufacturer’s instructions. All fractions were examined using the corresponding marker antibodies to confirm the success of fractionation.

### Polyclonal antibody preparation and immunoblot assay

The 241- to 750-bp region of the *ENB1* ORF corresponding 81–250 aa was cloned into the pGEX-4T-1 vector (Amersham Biosciences, Amersham, UK) for antibody production. The recombinant glutathione S-transferase (GST)-ENB1 was expressed in *Escherichia coli* Rosetta (DE3) cells by adding 0.5 mM of isopropylthio-β-galactoside (IPTG) when the optical density at 600 nm (OD_600_) of these cells reached 0.6. The GST-ENB1 was purified with the ÄKTA purification system (GE Healthcare, Chicago, IL, USA). The anti-ENB1 was produced in rabbits according to the standard protocols of ABclonal Technology.

Proteins extracted from developing endosperms were separated by sodium dodecyl sulfate–polyacrylamide gel electrophoresis (SDS–PAGE) and then transferred to a polyvinylidene difluoride (PVDF) membrane (Millipore, Burlington, MA, USA; cat. no. IPVH00010). Primary and secondary antibodies were used to recognize the target protein attached to the PVDF membrane. The signals were visualized with the SuperSignal West Dura Extended Duration Substrate (Thermo Fisher Scientific, Waltham, MA, USA; cat. no. 34075) at the Tanon 5200 chemiluminescence imaging system (Tanon Science & Technology, Shanghai, China). Purified anti-ENB1 antibody was used at 1:1,000, the anti-Tubulin antibody (Sigma-Aldrich, St. Louis, MO, USA; cat. no. T4026) was used at 1:5,000, the anti-Histone H3 antibody (Sigma-Aldrich, St. Louis, MO, USA; cat. no. H0164) was used at 1:5,000, the anti-H^+^ ATPase antibody (Agrisera, Vännäs, Sweden; cat. no. AS07260) was used at 1:5,000, and the anti-BIP antibody (Santa Cruz Biotechnology, Dallas, TX, USA; cat. no. sc-33757) was used at 1:1,000.

### Microscale thermophoresis assay

The CatD of ENB1 or ENB1^G780R^ (enb1), as described previously with minor modifications (Olek et al., 2014), was used for the UDP-Glc (Sigma-Aldrich, St. Louis, MO, USA; cat. no. U4625) binding assay. *ENB1* or *enb1 CatD* corresponding to the 1,015–2,523-bp region of *ENB1* or *enb1* ORF was cloned into the pCold-TF DNA Vector (Takara, Shiga, Japan) that contains an N-terminal His tag and a soluble tag of trigger factor (TF) chaperone. The recombinant His-TF-ENB1 CatD or His-TF-ENB1^G780R^ CatD was expressed in *E. coli* Rosetta (DE3) cells by adding 0.5 mM of IPTG when OD_600_ reached 0.8. Recombinant proteins were purified with the BeaverBeads IDA-Nickel (Beaver, Suzhou, China; cat. no. 70501-5) and then labeled with the Monolith Protein Labeling Kit RED-NHS 2nd Generation (NanoTemper, Munich, Germany; cat. no. MO-L011). The microscale thermophoresis assays were conducted using a Monolith NT.115 (NanoTemper, Munich, Germany) machine. The serial concentrations of UDP-Glc were titrated against 100 nM of His-TF-ENB1 CatD or His-TF-ENB1^G780R^ CatD protein in the optimized buffer (50 mM Tris–HCl, pH 7.4, 150-mM NaCl, 10-mM MgCl_2_, 0.05% Tween-20). Each protein was labeled 3 times for three independent tests. All data were analyzed using the MO. Affinity Analysis version 2.3 software. Relevant primer sequences are given in Supplemental Data Set 6.

### Cellulose quantification

Ten developing endosperms were dried and ground as one biological replicate, then 20 mg powder was used for preparing alcohol-insoluble residues (AIRs) of the CWs. De-starched AIR samples were produced as described previously (Li et al., 2009). Crystalline cellulose analysis was performed as described previously with minor modifications (Xiong et al., 2010). The samples were hydrolyzed in 2-M trifluoroacetic acid (TFA) at 121°C for 90 min. The remains after the TFA treatment were hydrolyzed in Updegraff reagent at 100°C for 30 min. The cooled samples were washed with acetone and hydrolyzed with 72% (v/v) sulfuric acid. The supernatant was used for measuring the cellulose amount by the anthrone assay (Updegraff, 1969).

### Kernel culture in vitro

Developing kernel in vitro culture was performed as described previously with minor modifications (Cheng and Chourey, 1999). Briefly, the ears were harvested at 4 DAP with the outer husks removed. The ears were sterilized with a 95% (v/v) alcohol spray and dried, and then immersed in 5% (v/v) bleach for 5 min in the laminar-flow hood. The ears were dissected into many two-row blocks with each block having six attached kernels, and then three kernels were removed from the alternating rows. Finally, the blocks with three attached kernels were placed on 100 × 25-mm plastic dishes with a solid Murashige and Skoog medium (PhytoTechnology Laboratories, Lenexa, KS, USA; cat. no. M519). The medium was supplemented with 10-mM glutamine, and 10-mM asparagine, 1-mg L^−1^ of 2, 4-dichlorophenoxyacetic acid. All medium was adjusted to pH 5.8 before the addition of 3% (m/v) or 15% (m/v) sucrose as well as the agar (5.5 g L^−1^). About 10 mg L^−1^ Streptomycin sulfate was filter-sterilized into all media after autoclaving. The kernels were cultured in a dark chamber at 28°C for 10 and 20 days.

### ^13^C-sucrose abundance analysis

The developing kernels were cultured in a nutrient solution including ^13^C-sucrose (Sigma-Aldrich, St. Louis, MO, USA; cat. no. 605417), which was prepared as described previously (Melkus et al., 2011). After culturing for 12 h in a dark chamber at 28°C, the kernels were rinsed 3 times with sterile H_2_O, and the embryos and the endosperms were isolated and pooled, respectively. Three biological replicates were made from three independent pooled samples (pooled endosperms or pooled embryos). The samples were freeze-dried, ground, and analyzed for the ^13^C/^12^C-isotope ratio using isotope ratio mass spectrometry Isoprime 100 (Elementar, Langenselbold, Germany). ^13^C-labeling abundance was calculated for the embryos and endosperms, respectively.

### Quantification of sugars by ion chromatography

Sugars (sucrose, glucose, and fructose) from developing endosperms were extracted as described previously with minor modifications (LeCLere, et al., 2010). Briefly, the weighed endosperms were added to 80% (v/v) ethanol and homogenized for 2 min. The extraction mixtures of ten endosperms were pooled as one biological replicate and heated to 85°C for 15 min. The samples were centrifuged at 16,100 *g* for 5 min at room temperature, then the ethanol supernatant was dried and re-dissolved in the sterile H_2_O for ion chromatography analysis. The sugar concentrations were determined using the Dionex ICS-5000+ chromatography system (Thermo Fisher Scientific, Waltham, MA, USA). The diluted samples were filtered through a 0.1-μm syringe filter and then separated on a Dionex CarboPac PA10 BioLC column (4 × 250 mm) (Thermo Fisher Scientific, Waltham, MA, USA). The flow rate was 1-mL min^−1^ and the column temperature was 35°C.

### RNA-Seq and RT-qPCR

Twenty 15 DAP endosperms of WT or *enb1* were pooled for extracting total RNA with an RNAprep Pure Plant Kit (TIANGEN, Beijing, China; cat. no. DP441). Three biological replicates were made from the kernels of three independent ears. cDNA libraries were constructed following the Illumina standard protocols and sequenced using an HiSeq X Ten platform (Illumina, San Diego, CA, USA) by Annoroad Gene Technology. Differential gene expression analysis was performed essentially as described previously (Trapnell et al., 2012). The clean reads were mapped to maize B73 RefGen_version 4.47 with TopHat (version 2.1.1). The transcripts were reconstructed and the gene expression levels were estimated with Cufflinks (version 2.1.1). The data were normalized as fragments per kilobase of exon per million fragments mapped. DEGs with a fold change and Q-value of the different expression above the threshold (fold change >2 and *Q* < 0.05) were identified as significant DEGs. The DEGs were functionally annotated and enriched using GO analysis (Tian et al., 2017).

RT-qPCR was conducted using SuperReal PreMix Plus (SYBR Green; TIANGEN, Beijing, China; cat. no. FP205) with an ABI 7500 Real-Time PCR System (Applied Biosystems, Waltham, MA, USA) or a CFX96 Real-Time PCR System (Bio-Rad, Hercules, CA, USA). Three independent RNA samples from the kernels of three F2 ears were used as biological replicates. The expression of maize *UBIQUITIN* (GenBank accession number: BT018032) was used as an internal control. Relevant primer sequences are given in Supplemental Data Set 6.

### Evans blue staining

Viability staining was performed as described previously with minor modifications (Young et al., 1997). Fresh WT and *enb1* kernels were obtained from the same segregating ear at 15 and 18 DAP, respectively. The kernels were cut along the longitudinal axis by hand, and the center sections were stained in 0.1% (w/v) Evans blue for 2 min. After washing the stained sections with water for 30 min, the consistently stained sections were observed using a light microscope SZX7 (Olympus, Tokyo, Japan).

### EMSA

The full-length ORF of *MRP1* was cloned into the pCold-TF DNA Vector (Takara, Shiga, Japan). Recombinant His-TF-MRP1 was expressed in *E. coli* BL21 (DE3) cells by adding 0.1 mM IPTG when OD_600_ reached 0.8. His-TF-MRP1 and His-TF (negative control) were purified with BeaverBeads IDA-Nickel (Beaver, Suzhou, China; cat. no. 70501-5) and used for EMSA. EMSA was performed as previously described with minor modifications (Li et al., 2015). Approximately 200 ng of purified His-TF-MRP1 and 5 ng 5′-biotin-labeled probes (WT probe or Mu probe harboring mutated TATCTA motif) were added to the reaction mixtures according to the standard protocol of the LightShift EMSA Optimization & Control Kit (Thermo Fisher Scientific, Waltham, MA, USA; cat. no. 20148X). Competition EMSA for the biotin-labeled probe was conducted by adding 50, 200-fold excess of unlabeled WT or Mu probes. Mutation EMSA for the biotin-labeled probe was conducted by mutating TATCTA motif. The samples were electrophoresed in a 6% native polyacrylamide gel. DNA and DNA–protein complexes were transferred to an Amersham Hybond-N+ membrane (GE Healthcare, Chicago, IL, USA; cat. no. RPN303B) and UV cross-linked. The biotin-labeled probes were detected using a Chemiluminescent Nucleic Acid Detection Module Kit (Thermo Fisher Scientific, Waltham, MA, USA; cat. no. 89880). Bands were visualized using the Tanon 5200 chemiluminescence imaging system (Tanon Science & Technology, Shanghai, China). Relevant primer sequences are given in Supplemental Data Set 6.

### Transient expression in maize protoplasts

The full-length ORF of *MRP1* was constructed into the pHB overexpression vector to generate the effector. The WT or mutated promoter of *ENB1* (–565 bp from the transcription start site) was constructed in the pGreen II 0800-LUC vector that contains the *firefly* *LUC* coding sequence to generate the reporter, respectively. The *Renilla* *LUC* (*REN*) gene driven by the CaMV 35S promoter in the pGreen II 0800-LUC vector was used as the internal control. Mesophyll protoplasts of maize leaves were isolated from 12-day-old etiolated W22 seedlings as described previously (Yoo et al., 2007). Released protoplasts were collected and then subjected to transformation using a polyethylene glycol-mediated plasmid delivery method (Yoo et al., 2007). The transfected protoplasts were treated with 1-mM sucrose and then cultured in the dark at 25°C for 12–16 h. The LUC and REN LUC activities of the protoplasts were measured using the Dual-LUC Reporter Assay System (Promega, Madison, WI, USA; cat. no. E1960) with a multimode reader Spark (TECAN). The ratio between LUC and REN activities was calculated as the relative LUC activity. Four biological replicates were made from four independent protoplasts. Relevant primer sequences are given in Supplemental Data Set 6.

### Statistical analysis

All Student’s *t* tests are given in Supplemental Data Set 7.

## Accession numbers

Sequence data from this article can be found in the GenBank/EMBL databases under the following accession numbers: CESA5/ENB1 (NP_001104955, Zm00001d034553); MRP1 (XP_008656502, Zm00001d010889); MN1 (NP_001105596, Zm00001d003776); SWEET4c (NP_001141590, Zm00001d015912); BAP2 (NP_001131993, Zm00001d049576); BETL9 (NP_001152629, Zm00001d041822); TCRR1 (NP_001288384, Zm00001d050200); SWEET4b (NP_001360983, Zm00001d015914); SWEET6a (NP_001149011, Zm00001d044421); SWEET16 (ONL97913, Zm00001d029098); SUT1 (NP_001104840, Zm00001d027854). RNA-Seq data that support the findings of this study have been deposited in the NCBI SRA database under the accession number PRJNA681735.

## Supplemental data

The following materials are available in the online version of this article.

**Supplemental Figure S1.** Phenotypic features of the maize *enb1* mutant.

**Supplemental Figure S2.** CRISPR/Cas9-based mutant of *ENB1*.

**Supplemental Figure S3.** Phylogenetic relationships and expression pattern of maize CESAs.

**Supplemental Figure S4.** mRNA ISH of the *ENB1* using the sense probe.

**Supplemental Figure S5.** Substrate binding assay of His-TF (negative control) to substrate UDP-Glc.

**Supplemental Figure S6.** *enb1* impairs the development of BETL cells.

**Supplemental Figure S7.** *enb1* reduces starch synthesis in the endosperm.

**Supplemental Figure S8.** *enb1* upregulates the expression of genes encoding hydrolase, sugar transporter, and ROS-related protein.

**Supplemental Figure S9.** Low sucrose supply downregulates the expression of genes involved in starch and auxin synthesis.

**Supplemental Figure S10.** *ENB1* overexpression enhances the endoreduplication of endosperm cells.

**Supplemental Table S1**. List of gene annotation information of all eight genes in the mapping interval.

**Supplemental File S1**. Alignment file used for the phylogenetic analysis shown in Supplemental Figure S3A.

**Supplemental File S2**. Newick format of the phylogenetic tree shown in Supplemental Figure S3A.

**Supplemental File S3**. Alignment file used for the phylogenetic analysis shown in Figure 3A.

**Supplemental File S4**. Newick format of the phylogenetic tree shown in Figure 3A.

**Supplemental Data Set 1**. Significantly DEGs of *enb1* endosperms compared with WT endosperms at 15 DAP.

**Supplemental Data Set 2**. GO terms of downregulated DEGs in *enb1* endosperms.

**Supplemental Data Set 3**. GO terms of upregulated DEGs in *enb1* endosperms.

**Supplemental Data Set 4**. Significantly DEGs of *enb1* embryos compared with WT embryos at 15 DAP.

**Supplemental Data Set 5**. GO terms of upregulated DEGs in *enb1* embryos.

**Supplemental Data Set 6**. List of primer sequences used in this study.

**Supplemental Data Set 7**. List of methods and parameters of statistics used in this study.

**Supplemental Movie S1**. Mobility of ENB1-EYFP particles.

**Supplemental Movie S2**. Observation of SWEET4c-EYFP fluorescence signals.

## Supplementary Material

koab312_Supplementary_Data

## Notes

R.S. conceived the project and designed the experiments. Q.W. and M.W. performed the experiments. All authors analyzed the data. Q.W. and R.S. wrote the manuscript.

The author responsible for distribution of materials integral to the findings presented in this article in accordance with the policy described in the Instructions for Authors (https://academic.oup.com/plcell) is: Rentao Song (rentaosong@cau.edu.cn).
