# Supplementary material for: ENB1 encodes a cellulose synthase 5 that directs synthesis of cell wall ingrowths in maize basal endosperm transfer cells
Source: Plant Cell. 2021 Dec 22;34(3):1054–74. doi: 10.1093/plcell/koab312 (PMC8894971; doi:10.1093/plcell/koab312)
Supplement: koab312_Supplementary_Data [file koab312_supplementary_data.zip › Supplemental Data.pdf]

A

Genotype	E1	E2	E3
WT	232	176	222
enb1	78	56	69
χ^2	0.004	0.092	0.25

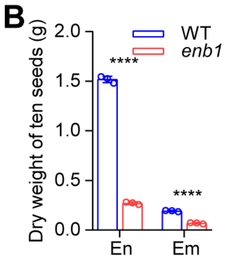

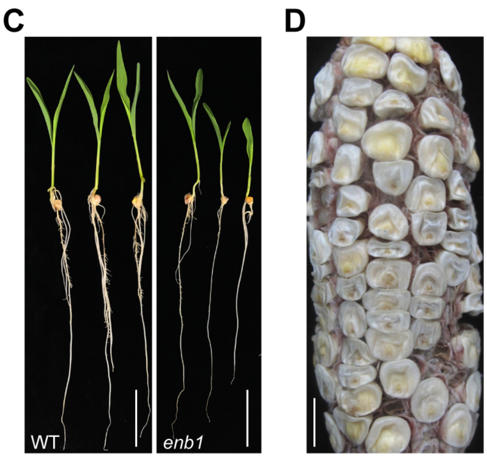

Supplemental Figure S1. Phenotypic features of the maize *enb1* mutant. (Supports Figure 1).
(**A**) Chi-square tests for the kernel phenotype from three independent F2 ears (E1, E2, and E3).
(**B**) Dry weight measurements of endosperm and embryo of WT and *enb1*. En, endosperm; Em, embryo. Data are mean ± SEM (n = 3 biologically independent samples). ****, P< 0.0001; Student's t-test.
(**C**) Phenotype of WT and *enb1* seedlings at 9 DAG. Bar = 5 cm.
(**D**) Phenotype of homozygous mature *enb1* ear. Bar = 1 cm.

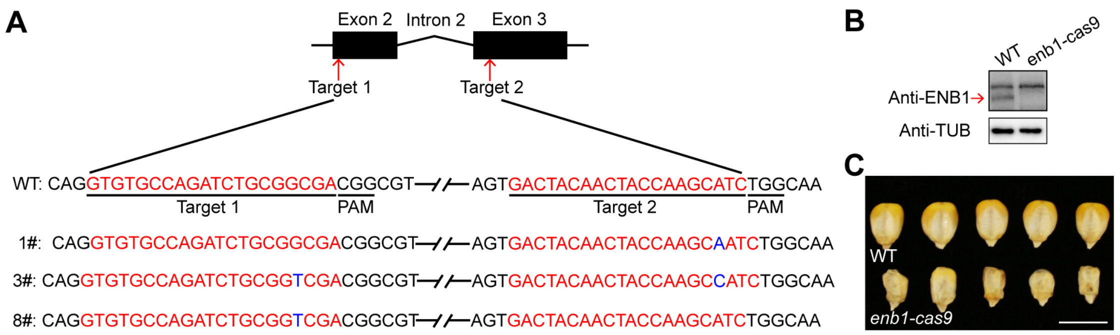

Supplemental Figure S2. CRISPR/Cas9-based mutant of *ENB1*. (Supports Figure 2).

(**A**) Diagram of CRISPR/Cas9 editing target sites in exon 2 and exon 3 of *ENB1*. Two targets are highlighted in red, PAM represents the protospacer adjacent motif. Inserted nucleotides are in blue.

(**B**) Protein accumulation of ENB1 in 15 DAP WT and *enb1-cas9* endosperms.

(**C**) Randomly selected mature kernels of WT and *enb1-cas9* in a segregated population. Bar = 1 cm.

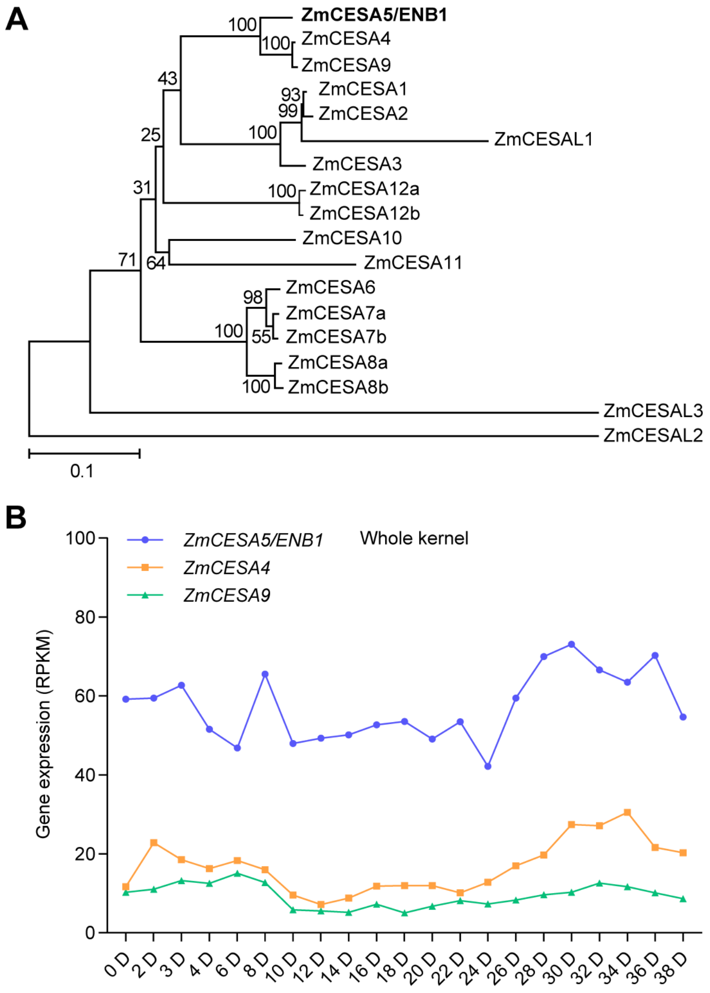

Supplemental Figure S3. Phylogenetic relationships and expression pattern of maize CESAs. (Supports Figure 3).

(**A**) Phylogenetic relationships of maize CESAs. These sequences were aligned by the MUSCLE method, and the phylogenetic tree was constructed using the neighbor-joining method in the MEGA-X software package. The numbers at the nodes represent the percentage of 1000 bootstraps. Scale bar, the average number of amino acid substitutions per site.

(**B**) Expression of *ZmCESA5/ENB1* and two closely related paralogs (*ZmCESA4* and *ZmCESA9*) in developing kernels (0 to 38 DAP). Data were collected from the Chen et al. (2014) gene expression dataset.

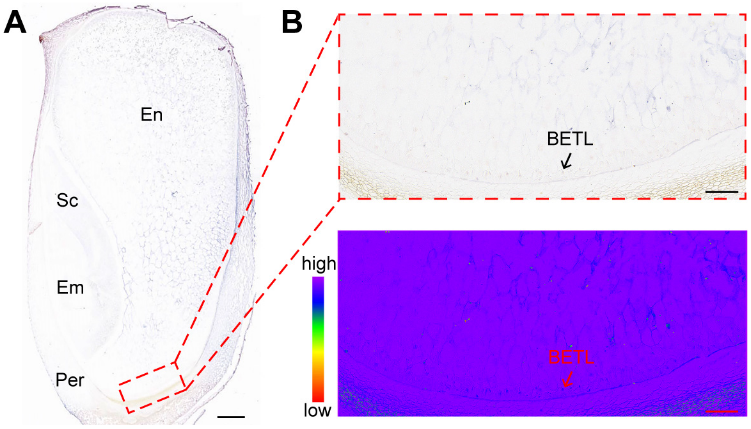

Supplemental Figure S4. mRNA *in situ* hybridization (ISH) of *ENB1* using the sense probe. (Supports Figure 3).

(A) and **(B)** As a negative control, 15 DAP W22 kernel sections were hybridized with an *ENB1* sense probe. The upper panel of **(B)** is the magnified section from the red dotted boxes of **(A)**. In **(B)**, the lower panel is a pseudo-color image according to the gray value of the upper panel (the lower the gray value, the more intense the ISH staining). Per, pericarp; En, endosperm; Em: embryo; Sc, scutellum. Bar = 500 μm (left) , 100 μm (right).

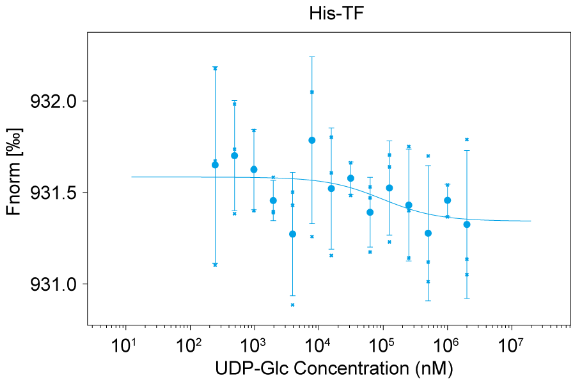

Supplemental Figure S5. Substrate binding assay of His-TF (negative control) to substrate UDP-Glc. (Supports Figure 4).

Fourteen dilutions of UDP-Glc were used for microscale thermophoresis measurements. Data are mean ± SD (n = 3 independent experiments).

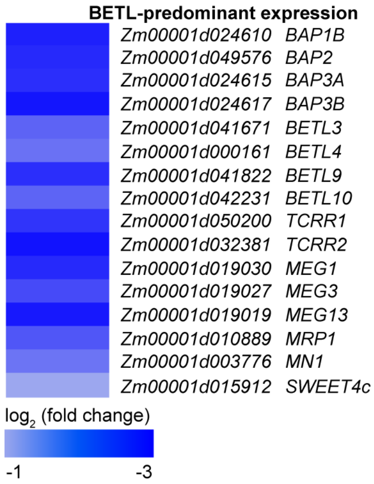

Supplemental Figure S6. *enb1* impairs the development of BETL cells. (Supports Figure 5). Heatmap depicting the $\log_2$ (fold change) of representative down-regulated BETL-predominately expressed genes in *enb1* endosperms.

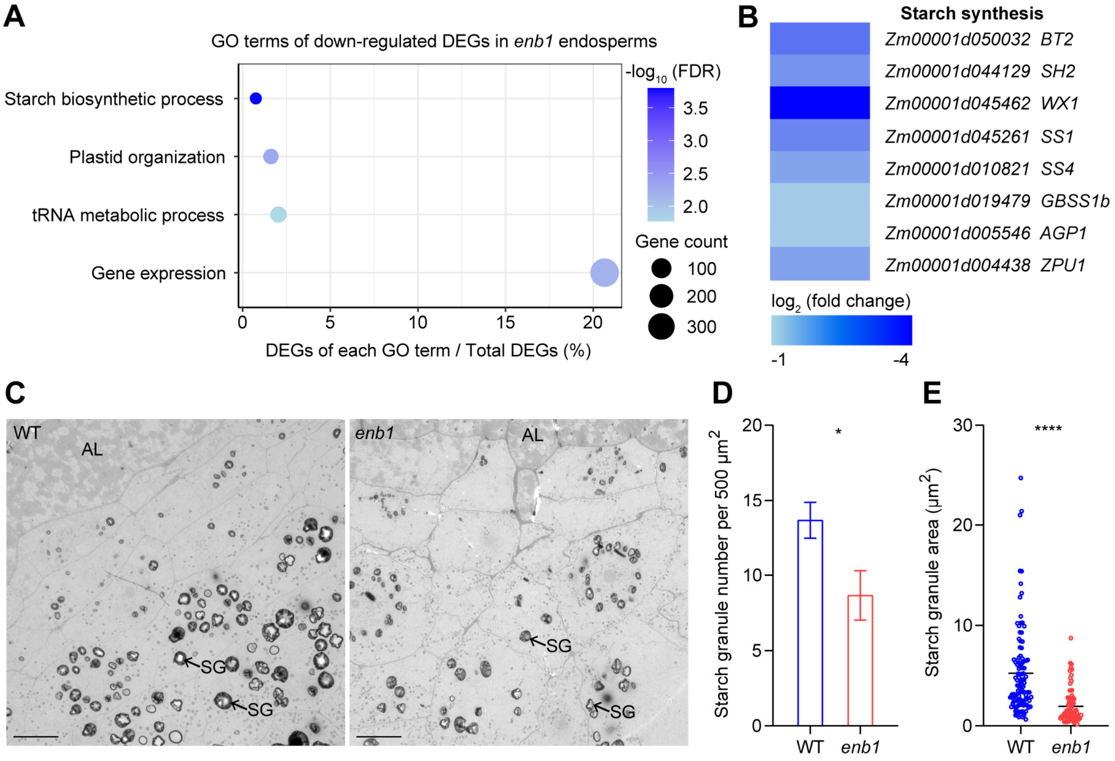

Supplemental Figure S7. *enb1* reduces starch synthesis in the endosperm. (Supports Figure 5).

(**A**) GO analysis of down-regulated DEGs in *enb1* endosperms. Circle sizes indicate DEGs numbers and the color gradients indicate enrichment significance.

(**B**) Heatmap depicting the $\log_2$ (fold change) of representative down-regulated DEGs associated with starch synthesis in *enb1* endosperms.

(**C**) TEM micrographs of 15 DAP WT and *enb1* endosperms. SG, starch granule; AL, aleurone layer. Bar = 10 μm.

(**D**) Estimation of starch granule number per 500 μm^2 of 15 DAP WT and *enb1* starchy endosperm cells near the aleurone layer. Data are mean ± SEM (n = 6 independent areas). *, P< 0.05; Student's t-test.

(**E**) Surface area of starch granules (SG) of 15 DAP WT and *enb1* starchy endosperm cells near the aleurone layer. Black lines indicate mean. n = 128 SGs in WT, n = 86 SGs in *enb1*. ****, P< 0.0001; Wilcoxon Rank Sum test.

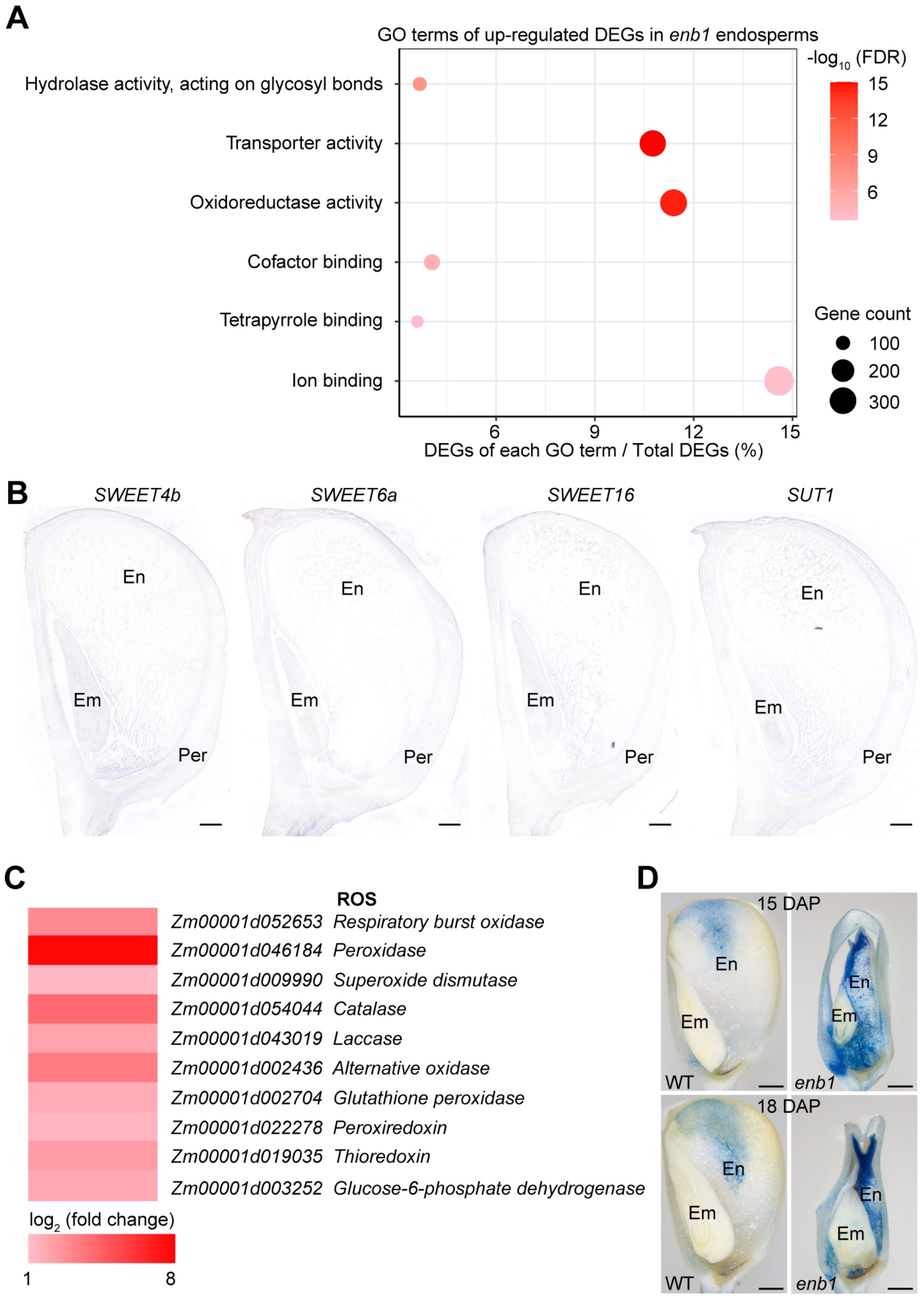

Supplemental Figure S8. *enb1* up-regulates the expression of genes encoding hydrolase, sugar transporter, and ROS-related protein. (Supports Figure 6).

(**A**) GO analysis of up-regulated DEGs in *enb1* endosperms. Circle sizes indicate DEGs numbers and the color gradients indicate enrichment significance.

(**B**) mRNA *in situ* hybridization of *SWEET*s and *SUT1*. As the negative controls, 15 DAP W22 kernel sections were hybridized with the sense probes of these genes. Per, pericarp; En, endosperm; Em, embryo. Bar = 500 μm.

(**C**) Heatmap depicting the $\log_2$ (fold change) of representative up-regulated ROS-related genes in *enb1* endosperms.

(**D**) Cell viability staining of the WT and *enb1* kernels at 15 and 18 DAP using the Evans blue. En, endosperm; Em, embryo. Bar = 1 mm.

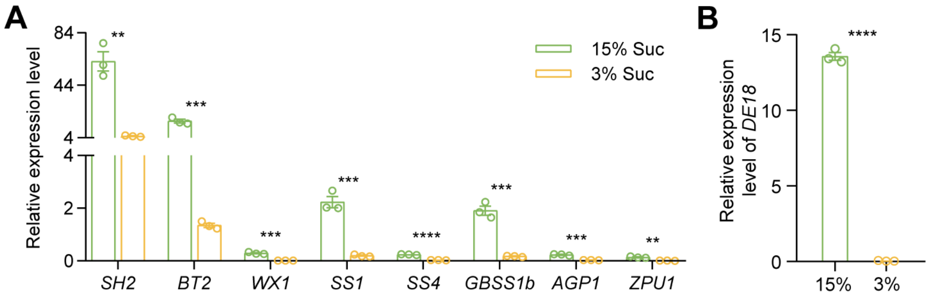

Supplemental Figure S9. Low sucrose supply down-regulates the expression of genes involved in starch and auxin synthesis. (Supports Figure 8).

Expression of starch (**A**) and auxin (**B**) synthesis genes of endosperms isolated from kernels cultured for 20 days in 15% or 3% sucrose. Data are mean ± SEM (n = 3 biologically independent samples). ****, P< 0.0001; ***, P< 0.001; **, P< 0.01; Student's t-test.

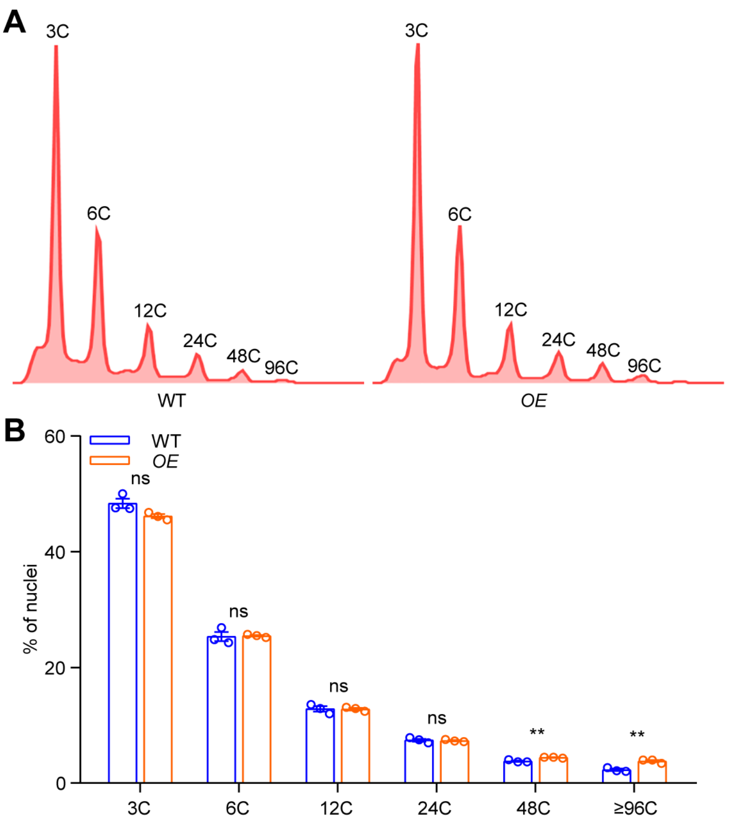

Supplemental Figure S10. *ENB1* overexpression enhances the endoreduplication of endosperm cells. (Supports Figure 10)

(**A**) Flow cytometric profile of WT and *OE* endosperms at 12 DAP. The C-value is indicated for each peak.

(**B**) Distribution of nuclear DNA, which was represented as a percentage of the total DNA in 12 DAP WT and *OE* endosperms. Data are mean ± SEM (n = 3 biologically independent samples). **, P< 0.01; ns, not significant; Student's t-test.

Supplemental Table S1. List of gene annotation information of all 8 genes in the mapping interval.

**Gene ID**	**Gene annotation**
Zm00001d034551	Polygalacturonase
Zm00001d034552	Polygalacturonase
Zm00001d034553	Cellulose synthase 5
Zm00001d034554	Cellulose synthase 4 (only 65 aa)
Zm00001d034555	Disease resistance protein RPM1
Zm00001d034556	Low confidence gene
Zm00001d034557	Low confidence gene
Zm00001d034558	Uncharacterized protein
