# Supplementary material for: ENB1 encodes a cellulose synthase 5 that directs synthesis of cell wall ingrowths in maize basal endosperm transfer cells
Source: Plant Cell. 2021 Dec 22;34(3):1054–74. doi: 10.1093/plcell/koab312 (PMC8894971; doi:10.1093/plcell/koab312)
Supplement: koab312_Supplementary_Data [file koab312_supplementary_data.zip › Supplemental Movie Legends.pdf]

Supplemental Movie S1. Mobility of ENB1-EYFP particles.
The ENB1-EYFP was transiently expressed in onion epidermal cells, then live cell imaging was performed using spinning-disk confocal microscopy (SDCM). Time-lapse images of ENB1-EYFP particles were collected with a 3-s interval for 100 frames, which were saved as movie format using ImageJ software.

Supplemental Movie S2. Observation of SWEET4c-EYFP fluorescence signals.
The SWEET4c-EYFP was transiently expressed in onion epidermal cells, then live cell imaging was performed using SDCM. Time-lapse images of SWEET4c-EYFP fluorescence signals were collected with a 3-s interval for 100 frames, which were saved as movie format using ImageJ software.
